# Localization of Biobotic Insects Using Low-Cost Inertial Measurement Units

**DOI:** 10.3390/s20164486

**Published:** 2020-08-11

**Authors:** Jeremy Cole, Alper Bozkurt, Edgar Lobaton

**Affiliations:** Department of Electrical and Computer Engineering, NC State University, Raleigh, NC 27695, USA; jacole@ncsu.edu (J.C.); aybozkur@ncsu.edu (A.B.)

**Keywords:** biobots, cyborg insects, inertial navigation, machine learning

## Abstract

Disaster robotics is a growing field that is concerned with the design and development of robots for disaster response and disaster recovery. These robots assist first responders by performing tasks that are impractical or impossible for humans. Unfortunately, current disaster robots usually lack the maneuverability to efficiently traverse these areas, which often necessitate extreme navigational capabilities, such as centimeter-scale clearance. Recent work has shown that it is possible to control the locomotion of insects such as the Madagascar hissing cockroach (*Gromphadorhina portentosa*) through bioelectrical stimulation of their neuro-mechanical system. This provides access to a novel agent that can traverse areas that are inaccessible to traditional robots. In this paper, we present a data-driven inertial navigation system that is capable of localizing cockroaches in areas where GPS is not available. We pose the navigation problem as a two-point boundary-value problem where the goal is to reconstruct a cockroach’s trajectory between the starting and ending states, which are assumed to be known. We validated our technique using nine trials that were conducted in a circular arena using a biobotic agent equipped with a thorax-mounted, low-cost inertial measurement unit. Results show that we can achieve centimeter-level accuracy. This is accomplished by estimating the cockroach’s velocity—using regression models that have been trained to estimate the speed and heading from the inertial signals themselves—and solving an optimization problem so that the boundary-value constraints are satisfied.

## 1. Introduction

Disasters are defined as discrete meteorological, geological, or man-made events that exceed local resources to respond and contain [1]. Disaster response is the phase of emergency management that is focused on saving the lives of those affected by the disaster and mitigating further damage by the disaster. Over the past 50 years, mankind has become increasingly urbanized, with roughly 55% of the human population living in urban areas [2]. As such, it is increasingly likely that disasters will occur in urban areas. This has led to the formation of specialized Urban Search and Rescue (USAR) teams that are capable of responding to a wide range of disasters in urban areas [3]. Time sensitivity and operation under harsh conditions are among the main challenges for search and rescue. USAR teams have been called upon to conduct operations in areas of extreme heat [4] or radiation [5], as well as environments containing explosive gases [6] or airborne pollutants such as carcinogens [7]. The aforementioned issues have birthed an entire discipline of field robotics, coined disaster robotics (or alternatively, search and rescue robotics) [8]. Disaster robotics is concerned with the design and deployment of robotic agents—whether they be ground, aerial, or marine—that are capable of addressing the challenges of disaster response.

USAR teams often use Unmanned Ground Vehicles (UGVs) to explore areas that would be impossible to rapidly, and safely, explore themselves. Existing robotic platforms can be used in areas where there are several meters of clearance; however, urban ruins can contain rubble piles or damaged buildings with voids that are several centimeters wide, with high tortuosity and verticality, and exhibiting a wide range of surface properties. Current technology is limited in its ability to miniaturize a robot to this scale while retaining enough mobility to traverse these environments. A potential solution to the mobility problem comes in the form of biologically-inspired robotics [9], a field of robotics that is interested in creating robots that mimic animal locomotion. Biomimetic modes of motion include legged locomotion (e.g., rHex [10] and VelociRoACH [11]) and serpentine locomotion (e.g., Active Scope Camera [12]). Research has also been conducted into creating grippers that mimic the adhesive behavior of insects and geckos (e.g., [13,14]). Though these methods are promising, it is still uncertain how they will be miniaturized to the centimeter scale while retaining their mobility across a wide range of surfaces.

**Figure 1:**
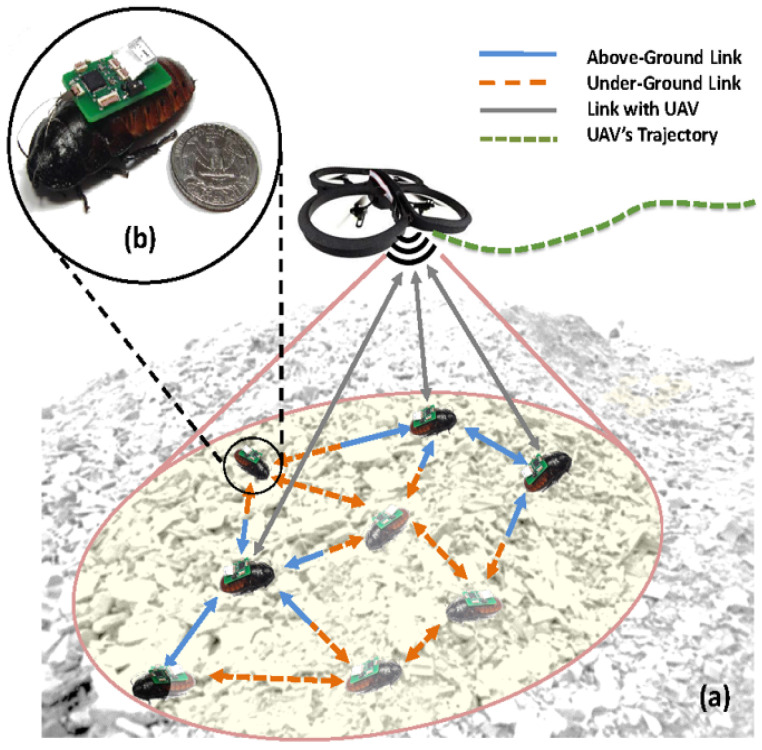
Diagram of a biobotic network: (**a**) Biobots serve as above/below ground agents and can be controlled either individually or collectively via a leader agent (in this case, a UAV). (**b**) Each biobot is several centimeters in length and a United States quarter dollar is shown for scale comparison [21].

Researchers [15,16,17] have shown that it is possible to remote control a Madagascar hissing cockroach (*Gromphadorhina portentosa*) via the bioelectrical stimulation of its neuro-mechanical system. These roaches grow to be approximately 60 mm long and 30 mm wide, with a payload capacity of approximately 15 g [15]. They use a combination of pretarsal claws and adhesive pads to cling to and move on a wide variety of surfaces [18], with top speeds of several cm/s. Their exoskeleton is a compliant structure, allowing them to fall from heights and squeeze under obstacles [19] without issue. Additionally, *G. portentosa* has the ability to survive days without water and weeks without food. This combination of features could make *G. portentosa* suitable for USAR teams in disaster response. As shown in the literature, these cockroaches can be outfitted with various electronic sensor payloads to be used for search, reconnaissance, and mapping tasks in urban ruins necessitating extreme mobility [20] (see Figure 1). These agents are referred to as biological robots, or biobots. Biobots can be used, both individually and in larger groups, to perform sensing tasks that are impractical or impossible to accomplish by other means. Sensing modalities may include microphone arrays for two-way audio, environmental sensors such as temperature and gas monitors, and cameras or infrared sensors for video feed. Each biobot has wireless capabilities, and special sensor payloads can be fabricated so that biobots can act as mobile repeaters to improve communication reliability.

USAR scenarios present substantial challenges for localization and mapping. Traditional techniques for localization that rely on Global Positioning Systems (GPS) are not feasible as GPS signals may be unavailable under the rubble. Furthermore, environmental hazards—such as fire and smoke —make it so that many commonly used ranging techniques (e.g., LIDAR/RADAR) become unreliable. Even vision-based techniques can fail in the presence of dirt, mud, and debris. Dirafzoon et al. [22] have recently proposed a solution for mapping that is based on Topological Data Analysis. This method generates a coordinate-free map of an environment using a group of biobots by keeping track of when they come into close proximity with one another. This map can be used to track the connectivity of a group of biobots, and given sufficient coverage of an area, it can also provide a coarse estimate of what obstacles (e.g., voids in the environment or physical impediments) are present. Two limitations of this approach are: first, the map does not contain accurate metric information—i.e., it cannot give responders the location of point of interest; secondly, the algorithm requires a large number of biobotic agents to be deployed in an area, which may not always be feasible. The two main contributions of this work are as follows:

- Development of a data-driven model for determining the speed of a biobotic agent (*G. portentosa*) based solely on inertial signals obtained from a thorax-mounted Inertial Measurement Unit (IMU).
- Design and verification of an inertial navigation system that is capable of estimating the pose of *G. portentosa* without the aid of additional sensing modalities.

Our navigation system requires minimal sensing modalities and will function with a single biobotic agent, eliminating the need for the biobot to use high-bandwidth/high-power sensors, such as cameras, for navigation.

The remainder of the paper is as follows: Section 2 introduces the topic of inertial navigation as well as work that is related to our system; Section 3 provides an overview and mathematical formulation of our navigation system; Section 4 describes the details of our navigation system; Section 5 details the experimental setup used for analysis and validation; Section 6 documents the performance of our navigation system; Section 7 concludes the paper and discusses ongoing and future work.

## 2. Related Work

A localization system that relies purely on inertial signals is known as an Inertial Navigation System (INS) [23]. Furthermore, inertial signals are often used in conjunction with other sensing modalities to create integrated navigation systems that are capable of localization. A brief review of integrated navigation systems, focusing on those that use IMUs, is presented next.

The goal of a navigation system is to estimate the position and/or orientation of an agent. When both position and orientation are tracked, the resultant system is said to estimate the pose of an agent. In the context of this paper, we will refer to pose estimation as ’localization’. There are a variety of sensing modalities that can be combined with inertial signals to localize an agent, one of the most common being Global Navigation Satellite Systems (GNSS) such as GPS. Systems combining both visual and inertial data are also becoming more common due to a combination of improved on-board processing capabilities, lower camera costs, and increased camera resolution (e.g., [24,25,26,27]). Other common sensors used to supplement inertial navigation systems include range finders such as LIDAR [28,29,30], RADAR [31,32], and SONAR (typically for underwater applications) [33,34].

The position and orientation of an agent can be obtained through double integration of the accelerometer and single integration of the gyroscope signals; however, IMU sensor noise renders these results unusuable after a short period of time for all but the most precise (i.e., expensive) IMUs. As such, navigation systems that rely solely on integrating the inertial signals are rare, and navigation is usually achieved by supplementing inertial signals with data obtained via additional sensing modalities. Usually, the sensor modalities are integrated using a Kalman Filter. Two common INS/GNSS frameworks are the ’loosely-coupled’ and ’tightly-coupled’ approaches [23], which use GNSS position/velocity and GNSS psuedo-range/psuedo-range rate, respectively. When GNSS is unavailable, as is the case for our application, then supplemental sensors, such as those listed in the previous paragraph, can be used for position and/or velocity estimation.

Pedestrian Dead Reckoning (PDR) is a particular application of inertial navigation where IMUs are used in a novel way [35]. The idea behind PDR is to use the inertial signals to determine a person’s stride by keeping track of when the feet hit the ground, thus limiting error growth by providing a measure of position/orientation displacement without the need to integrate the inertial signals themselves. The event pertaining to when a foot hits the ground, known as a zero-velocity update, can be tracked using an analytical [36,37] or a data-driven model [38,39,40]. Additionally, the stride lengths themselves can be determined analytically [41] or via a data-driven approach [42]. Unfortunately, many agents do not exhibit distinctive (and consistent) events such as zero-velocity updates. This is the root cause of the difficulty of inertial navigation in GNSS-denied areas—a lack of sensors and/or events that can be used to reduce the growth of pose error that is caused by noisy IMU signals [43]. As a result, machine learning techniques have been leveraged to learn position and velocity models as an alternative to deriving them from first principles. The effect of the IMU noise is mitigated since the noisy IMU signals are integrated into the model itself. The downside to this approach is that navigation accuracy is directly dependent on the data used to train the model(s). If the data used to train the model is dissimilar to the data obtained in the field, then the learned model(s) will provide poor approximations. Nevertheless, machine learning techniques have been successfully demonstrated for inertial navigation in areas where GNSS is unavailable.

Most inertial navigation systems track the position, velocity, and orientation, as well as the bias terms on the accelerometers and gyroscopes. These states comprise the traditional 15-state inertial navigation system, and it is these states that are estimated via data-derived models. Early examples of position/velocity models can be seen in [44,45]. In both papers, the target application was land vehicle navigation. The novelty of the papers came from using the GPS signal as the ground truth for training two neural networks that would be responsible for position and velocity estimation when GPS was unavailable. Under this framework, the vehicle used an INS/GPS system when GPS was available and switched to using the neural networks when GPS was unavailable. The position displacements were estimated using a neural network that took the INS estimates of velocity and orientation as inputs. In [44], the velocity estimation neural network took the INS velocity estimate and time information as inputs, whereas [45] only used the INS velocity estimate as input. Other authors have also taken the approach of using INS estimates to learn models for position and velocity estimation (e.g., [46,47]); however, the need to use INS estimates creates a potential problem—if GPS is lost for an extended period of time, then the navigation system will degrade in performance since the INS estimates will become increasingly erroneous. In [44], this problem was mitigated by creating a variant of their system that used the output of the velocity estimation neural network as input to the position estimation neural network instead of the INS velocity estimates. In [47], this issue was resolved by combining random forest regression with Principal Component Regression (PCR) [48]. Other authors have proposed using windowed inertial signals for pose estimation, thus avoiding this particular issue. Windowed approaches can be broken into two categories: models that use Long Short Term Memory (LSTM) neural networks [49,50,51,52] and models that use Convolutional Neural Networks (CNN) [38,53].

Some authors have created learning models that combine both LSTM and CNN approaches (e.g., [54]) while others have favored using ensemble learning methods in lieu of neural networks [55,56]. The majority of the models in the literature involve position or velocity estimation; however, these are not the only quantities that can be estimated. Orientation [50,54] and speed [38] can be estimated and the noise parameters for Kalman Filter frameworks can be learned as well [53].

There are two competing paradigms on how learned models should be incorporated into a navigation system: end-to-end frameworks and pseudo-measurements. The idea behind the end-to-end framework is that a learned model is sufficient to output the pose of an agent given its inertial signals. Systems utilizing the end-to-end paradigm tend to incorporate deep neural networks involving CNNs or LSTMs [50,51,54,57]. The primary benefit of an end-to-end framework is the ability to implicitly model the relationship between the agent’s egomotion (measured by the inertial signals) and its pose. As such, it becomes possible to create navigation systems using lower quality IMUs. The major downside of this approach is that the accuracy of these models is highly dependent on the data used to train them. Proponents of the psuedo-measurement approach argue that the best way to incorporate learned models is by adding them as additional measurements to an existing navigation system (e.g., a Kalman filter) [38,49,52,53,55,58]. The benefit of this approach is that the existing navigation system is augmented rather than replaced; however, the challenge of this approach comes from determining the details of how the learned model(s) will be integrated into the existing system. A middle ground approach has also been used in the literature, with the idea being to use the original navigation system when possible and the learned model(s) only when necessary [44,45,46,47,56]. A list of current trends and challenges in integrated navigation systems can be found in [59,60].

Our target application, navigation of centimeter-scale rubble stacks using biobotic agents, is a form of terrestrial navigation in a GNSS-denied environment. It shares similarities to the pedestrian and automobile localization problems that are commonly seen in the literature; however, there are two key distinctions: first, there are no zero-velocity events that occur with guaranteed regularity, and secondly, biobotic agents frequently change both their speed and their direction. To resolve these issues, we developed an inertial navigation system that utilizes regression models for estimating speed and heading. Speed regression was chosen as an alternative to velocity regression to simplify the training process. Other papers, such as [38], estimate speed for zero-velocity detection; however, these papers are concerned with determining if an agent is moving (i.e., a classification problem), whereas we are interested in how fast an agent is moving. Our algorithm computes heading by using a regression model to estimate the heading correction that must be applied to headings that are computed by an Attitude and Heading Reference System (AHRS). This idea of using a data-driven model to correct an INS output is similar to [55]; however, in that paper, the authors developed a model for determining position error. Although our speed and heading models use windowed inertial signals, similar to many of the papers listed in the preceding paragraphs, we explicitly extract the features from the inertial signals [61] whereas other approaches, such as [50,54], do this implicitly. Our models use random forests to avoid the overfitting issues that are commonly seen in neural networks. Our approach of using random forests is similar to [46]; however, that paper proposed a navigation system that took INS velocities as input and returned position displacements as output. We incorporate the speed and heading models into our navigation system by using them to solve a two-point boundary-value problem.

## 3. Problem Formulation

**Figure 2:**
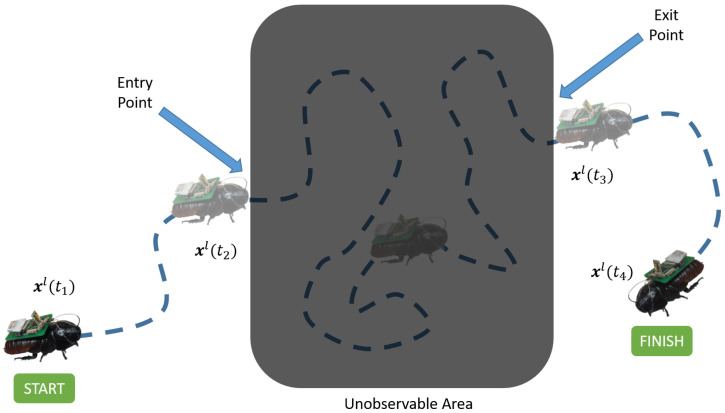
Problem Description: Suppose we want to track the trajectory of an agent over time. The agent can be observed during the time intervals $\left[t_{1},t_{2}\right]$ and $\left[t_{3},t_{4}\right]$; however, the agent is not observable during the time interval $\left(t_{2},t_{3}\right)$. As such, the agent’s state is unknown during this time interval. The goal of our algorithm is to estimate the agent’s state during the interval $\left(t_{2},t_{3}\right)$ so that the trajectory over the interval $\left[t_{1},t_{4}\right]$ can be reconstructed. See Section 3 for a description of the agent’s state, $x^{l}$.

Consider the following scenario, illustrated in Figure 2: a USAR team needs to search a target area that is not easily accessible via conventional tools (e.g., a rubble stack exhibiting high tortuosity and centimeter-scale clearance). The team deploys a biobotic agent into the target area, where it explores the environment while simultaneously collecting pertinent sensor data. Once the biobot enters the target area, its pose is no longer observable; however, the biobot will eventually leave the target area, whereby its pose will once again be observable. The goal is to reconstruct the biobot’s trajectory using inertial data so that any signals of interest can be localized.

Our biobots use low-cost IMUs to decrease unit cost and increase scalability. These IMUs have noisy gyroscope signals that make it difficult to accurately estimate the orientation of the biobot. As such, the gyroscope signals must be supplemented with additional information to limit the error growth of the orientation. We chose to use the direction of gravity [62] as the supplemental information. A downside to this approach is that it necessitates an algorithm for determining the direction of gravity in the body frame of the biobot. Ordinarily, this process would be accomplished using an orientation estimate that is obtained from integrating the gyroscopes; however, this strategy is not viable due to sensor noise. To avoid this issue, we restrict the agent to 2D planar environments so that the direction of gravity in the body frame is known, and we leave the extension to 3D for future work.

The trajectory estimation problem can be formulated as a nonlinear two-point boundary-value problem [63,64], where the biobot’s pose at both the entry (start state) and exit (final state) points are known. In this boundary-value problem, the objective is to find an optimal state trajectory between the start and end pose. Optimality is measured by how well the reconstructed state trajectory matches the estimated speeds and headings that are obtained from the IMU mounted on the biobot.

For our application, we define a local tangent frame, *l*, and use it as both the reference and resolving frames of our navigation system, where l uses Cartesian coordinates. Additionally, we define the body frame (denoted by *b*) to be centered on the IMU that is mounted to the body of the biobotic agent itself, with origin $r_{lb}^{l}$. Note that the subscript of the term $r_{lb}^{l}$ means “frame *b* with respect to (w.r.t.) frame *l*”, and the superscript means “resolved using frame *l*”. The coordinate frames are illustrated in Figure 8.

**Figure 3:**
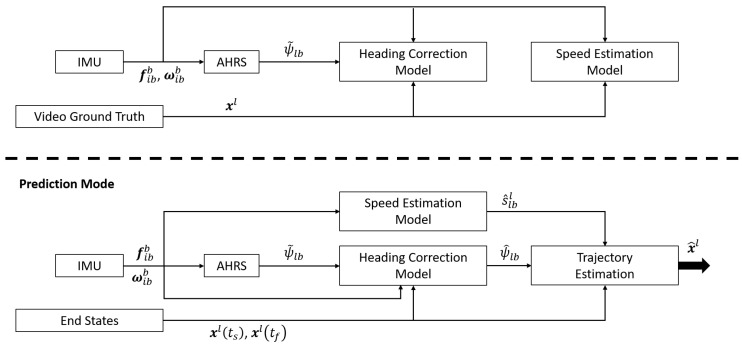
System Pipeline: The algorithm is broken into two modes of operation: Training Mode and Prediction Mode. In **training mode**, inertial signals are combined with video ground truth data to train regression models that can be used to estimate the speed and heading of the agent. In **prediction mode**, the trained speed/heading models are used to estimate the speed and heading from the inertial signal input. The estimated speeds/headings are then combined to reconstruct the trajectory of the agent. $f_{ib}^{b}$ and $\omega_{ib}^{b}$ denote the specific force measured by the accelerometer and the angular rate measured by the gyroscope, respectively—frame *i* denotes the Earth-centered inertial frame. ${\hat{s}}_{lb}^{l}$ is the biobot’s estimated speed, ${\tilde{\psi }}_{lb}$ is the heading estimate obtained from the AHRS, and ${\hat{\psi }}_{lb}$ is the biobot’s estimated heading after it has been corrected.

We define the biobot’s state, $x^{l}\left(t\right)$, to be its position, speed, and heading:

(1) $$x^{l}\left(t\right)={\left[r_{lb}^{l}\left(t\right),s_{lb}^{l}\left(t\right),\psi_{lb}\left(t\right)\right]}^{T}$$

where $r_{lb}^{l}=\left(x_{lb}^{l},y_{lb}^{l}\right)$, $s_{lb}^{l}=\sqrt{{\left({\dot{x}}_{lb}^{l}\right)}^{2}+{\left({\dot{y}}_{lb}^{l}\right)}^{2}}$, $\psi_{lb}$, denote the biobot’s position, speed, and heading, respectively. We assume that the biobotic agent always moves in the direction that it is facing. Under this assumption, we can recover the biobot’s velocity, $v_{lb}^{l}={\dot{r}}_{lb}^{l}=\left({\dot{x}}_{lb}^{l},{\dot{y}}_{lb}^{l}\right)$, by combining its speed and heading: ${\dot{x}}_{lb}^{l}=s_{lb}^{l}\cdot \cos\left(\psi_{lb}\right)$ and ${\dot{y}}_{lb}^{l}=s_{lb}^{l}\cdot \sin\left(\psi_{lb}\right)$. Note that this model is very similar to the Dubins car [65] and Reeds–Shepp car [66] models that are commonly used in robotics; the difference is that those models use speed and angular rate as inputs, whereas our model defines speed to be a state and uses specific force and angular rate as inputs (see Figure 3), denoted as $f_{ib}^{b}$ and $\omega_{ib}^{b}$, respectively.

We represent the biobot’s true trajectory as a smooth mapping in $R^{4}$, $x^{l}\left(t\right):\left[0,t_{f}\right]\to R^{4}$, where $t_{f}$ denotes the biobot’s exit time. Our goal is to find a reconstruction of $x^{l}$, ${\hat{x}}^{l}\left(t;\theta \right):\left[0,t_{f}\right]\to R^{4}$, where θ denotes the set of parameters that govern ${\hat{x}}^{l}$. The optimal parameters are obtained by minimizing the following cost functional:

(2) $$\begin{array}{cc} \; & J\left(\theta \right)=\int_{t_{s}}^{t_{f}}\left|\right|x^{l}\left(t\right)-{\hat{x}}^{l}\left(t\right){\left|\right|}^{2}\;dt\\ \; & s.t.\;{\hat{x}}^{l}\left(t_{s}\right)=x^{l}\left(t_{s}\right)\;,\;{\hat{x}}^{l}\left(t_{f}\right)=x^{l}\left(t_{f}\right) \end{array}$$

subject to the boundary conditions, where $t_{s}$ and $t_{f}$ denote the entry and exit times, respectively. We pose this problem as a supervised machine learning problem, where the objective is to minimize Equation (2) by training a model that is capable of generating ${\hat{x}}^{l}\left(t\right)$ using inertial signals. The ground truth values of $x^{l}\left(t\right)$ are obtained from video footage of the biobot.

## 4. Methodology

The goal of our navigation system is to estimate a biobot’s trajectory during time intervals where it cannot be observed. We assume that the biobot’s state is known at the beginning and end of these time intervals, and use the inertial signals obtained from an IMU mounted on the biobot to generate a curve that best approximates the biobot’s trajectory. Our algorithm uses machine learning to accomplish this goal and the system pipeline is shown in Figure 3.

The models used in the algorithm are trained via supervised learning. As such, there are two phases to our algorithm: Training Mode and Prediction Mode. In training mode, features are extracted from the inertial signals and used to generate regression models for estimating the speed of the biobot and correcting the heading that is obtained from an Attitude and Heading Reference System (AHRS). In prediction mode, these two models are used to estimate the biobot’s trajectory.

This section provides the details necessary to implement our algorithm, and is broken down into subsections that correspond to the modules shown in Figure 3. Additionally, all models were implemented using the MATLAB *Statistics and Machine Learning* toolbox [67].

### 4.1. Attitude and Heading Reference System (AHRS)

An AHRS is a partial INS that only tracks orientation. These systems are often used to supplement gyroscopes that are too noisy to be used as standalone systems for computing orientation. Due to the noise on our gyroscopes, we use the Madgwick Filter [62], an AHRS commonly used in the robotics community, to compute the biobot’s orientation. The Madgwick Filter is a complementary filter that combines gyroscope integration, accelerometer leveling, and magnetic heading to produce an accurate estimate of orientation. Furthermore, the Madgwick Filter generates an orientation estimate for each IMU sample that is given as input. Since our application is prone to magnetic interference, we do not use the magnetic heading component of the Madgwick Filter. The Madgwick Filter and the ramifications of excluding the magnetic heading component from it are elaborated upon next, starting with the filter’s cost function:

(3) $$f\left({\hat{q}}_{l}^{b},g_{b}^{l},{\hat{g}}_{b}^{b}\right)={\hat{q}}_{l}^{b†}\circ g_{b}^{l}\circ {\hat{q}}_{l}^{b}-{\hat{g}}_{b}^{b}$$

where ${\hat{q}}_{l}^{b}$, $g_{b}^{l}$, and ${\hat{g}}_{b}^{b}$, denote the estimated orientation (in quaternion form) of the body w.r.t. the local tangent reference frame; direction of the body’s acceleration due to gravity (i.e., a unit vector), resolved in the local tangent reference frame; and the estimated direction of the body’s acceleration due to gravity, resolved in the body frame, respectively. Note that ${}^{†}$ and ∘ denote the quaternion conjugate and quaternion product, respectively. The interested reader can learn more about using quaternions as rotation operators in [68].

The idea behind Equation (3) is that the correct orientation estimate will be the orientation that minimizes the difference between the direction of the acceleration due to gravity resolved in the reference frame (in quaternion form), $g_{b}^{l}={\left[0,0,0,-1\right]}^{T}$, and the direction of the acceleration due to gravity resolved in the body frame. The underlying assumption of Equation (3) is that there exists a means by which ${\hat{g}}_{b}^{b}$ can be estimated. As mentioned in Section 3, we assume that the biobot is operating on the plane, and under this assumption, ${\hat{g}}_{b}^{b}=g_{b}^{l}$. The gradient of Equation (3) w.r.t. $q_{l}^{b}$, denoted as $\nabla f(q_{l}^{b})$, is used to update the orientation of the biobot, as follows:

(4) $$\begin{array}{cc} \; & {\hat{q}}_{l}^{b}\left(+\right)={\hat{q}}_{l}^{b}\left(-\right)+{\dot{q}}_{l}^{b}\left(+\right)\Delta t\\ \; & {\dot{q}}_{l}^{b}\left(+\right)={\dot{q}}_{l}^{b}\left(-\right)-\beta \frac{\nabla f({\hat{q}}_{l}^{b})}{\left||\nabla f({\hat{q}}_{l}^{b})|\right|}\\ \; & {\dot{q}}_{l}^{b}\left(-\right)=\frac{1}{2}\cdot {\hat{q}}_{l}^{b}\left(-\right)\circ {\left[0,\omega_{ib}^{b}\right]}^{T} \end{array}$$

where Δt denotes the sampling interval of the IMU, β is the gain of the filter, and (-) and (+) are used to designate whether a term has been computed *before* or *after* the update, respectively. The derivation of Equation (4) can be found in [62]. Since the biobot is restricted to the plane, we do not need to worry about singular points in Euler Angle sequences. As such, we extract the estimated heading of the biobot from the the Madgwick Filter, ${\tilde{\psi }}_{lb}$, by converting the quaternion orientation output to an extrinsic ZYX Euler Angle Sequence and storing the rotation around the +Z axis. The details on converting between quaternions and Euler Angles can be found in [68], Chapter 7.

As mentioned previously, the Madgwick filter has a third component to it that involves magnetic heading. Specifically, that term’s purpose is to create a unique orientation fix by using the direction of the Magnetic North Pole as an orthogonal direction to the direction of gravity. Since we cannot determine Magnetic North due to the magnetic interference that is likely present in our application, we can only restrict the orientation to a plane that is orthogonal to the estimated direction of gravity, ${\hat{g}}_{b}^{b}$. As such, the orientation generated by our AHRS will drift over time. This drift is caused by the gyroscope error and grows linearly in time, as shown in Figure 5 and discussed in Section 4.4.1.

### 4.2. Feature Extraction

Inertial Measurement Units produce specific force and angular rate readings, ${\left[f_{ib}^{b},\omega_{ib}^{b}\right]}^{T}$, at a specified sampling rate. Authors commonly use these inertial signals in their direct form (i.e., specific force/angular rate) or integrated form (e.g., velocity/orientation) when adding machine learning to an INS. Our speed estimation (Section 4.3) and heading correction (Section 4.4) models use time-domain features extracted from windowed inertial signals. This section describes the process of generating the inputs to our models from the calibrated inertial signals themselves.

Our model is trained using the dataset, $D:=\left[{\left\{d\left(\tau_{k}\right),s_{lb}^{l}\left(\tau_{k}\right),\psi_{lb}\left(\tau_{k}\right)\right\}}_{k=1..n}\right]$, where *n* denotes the number of data points in the dataset. The kth feature vector is denoted as $d(\tau_{k})$, where $\tau_{k}$ denotes the timestamp associated with the kth data point. Henceforth, “IMU sample” will refer to the IMU readings themselves, and “data point” will refer to the elements of D, unless explicitly stated otherwise.

Each data point is computed from a window of inertial data. We use a one-second sliding window with 50% overlap. This particular configuration was chosen based on empirical evidence that was shown in [61]. The goal of that paper was to recognize when biobots were exhibiting various motion-based activities using inertial signals obtained from an IMU mounted on their thorax. Speed regression and heading correction are also motion-based, hence we chose this particular configuration for the sliding window.

Each data point is timestamped using the timestamp of the first video frame in the window. The ground truth speed and heading that are associated with each data point are obtained via an algorithm that corrects the ground truth video frames so that the ground truth speeds and headings integrate to match the ground truth positions. This corrective algorithm is detailed in Section 4.6.

Each feature vector consists of 60 time-domain features shown in Table 1 that are extracted from the windowed IMU data. These features are commonly used for activity recognition using wearable sensors and were shown in [61] to also be useful for classifying motion-based activities for biobots. We also normalize the features to have zero mean and unit variance—this is commonly done to prevent features from having undue influence due to their relative magnitude to other features. By extracting features from the windowed IMU data, we reduce the dimensionality of our model input to 60, irrespective of window size. Furthermore, the extracted features increase our models’ robustness to noise and reduce their susceptibility to spurious IMU readings (e.g., outliers and/or missing data).

### 4.3. Speed Estimation Model

The goal of our speed estimation model is to estimate the speed of the biobot, ${\hat{s}}_{lb}^{l}$, using feature vectors created from windowed inertial data. There are two components to the speed estimation model: a classification model that can detect when the biobot is stationary, denoted as $M_{z}$, and a regression model that can estimate the speed of the biobot when it is not stationary, denoted as $M_{s}$. Explicitly, the structure of the speed estimation model is given by this equation:

(5) $${\hat{s}}_{lb}^{l}\left(\tau_{k}\right)=\left\{\begin{array}{cc} 0 & ,\;M_{z}\left(d\left(\tau_{k}\right)\right)=1\\ M_{s}\left(d\left(\tau_{k}\right)\right) & ,\;M_{z}\left(d\left(\tau_{k}\right)\right)=0 \end{array}\right.$$

where $\tau_{k}$ denotes the timestamp of the kth feature vector, as discussed in Section 4.2.

#### 4.3.1. Speed Regression

Biobots (*G. portentosa*) exhibit a tripod gait and preliminary analysis [69] has shown that it is possible to directly estimate a biobot’s speed from its inertial signals, as opposed to integrating the signals over time, as a result of the wobbling motion that is induced by the tripod gait. Using these findings, we designed $M_{s}:R^{F}\to R$, i.e., ${\hat{s}}_{lb}^{l}\left(\tau_{k}\right):=M_{s}\left(d\left(\tau_{k}\right)\right)$, where *F* denotes the number of features; $M_{s}$ is only used when the biobot is moving, as described in Equation (5).

**Figure 4:**
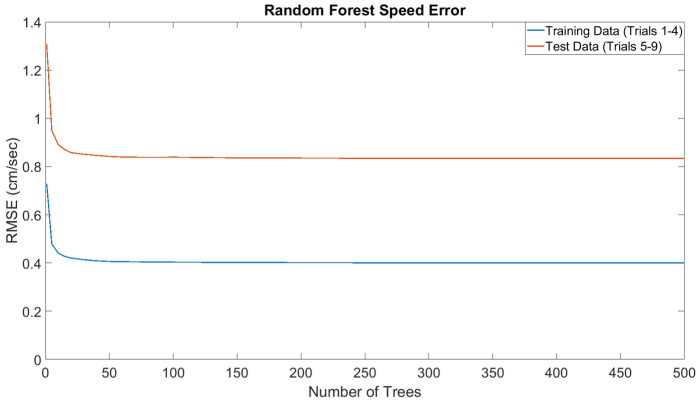
Analysis on Number of Trees: The Root Mean Square Error (RMSE) of the speed of the biobot is shown as a function of the number of trees in the random forest speed regression model, $M_{s}$. We use 100 trees for our speed regression model because the RMSE does not decrease if additional trees are added. Four trials were used to train the random forest regression model and the errors are reported for both the training data and the test data. Each trial is approximately 30 min long.

Ordinarily, speed is estimated by integrating the accelerations that are extracted from the IMU’s specific force readings; however, this is not feasible for low-cost IMUs because sensor noise renders these values unusable after a brief period of time. The error characteristics of our IMU (see Table 5) place it into this category. Furthermore, this issue is exacerbated by the lack of consistent measurements (e.g., zero-velocity updates) that can be used to curtail error growth, thus limiting our ability to apply traditional INS frameworks such as Kalman Filters. Fortunately, by using $M_{s}$, the biobot’s speed can directly estimated from its inertial signals, thus eliminating the linear error growth over time that occurs when obtaining the speed from integrating the acceleration. We realized $M_{s}$ using a random forest [70] of regression decision trees [71,72]. Specifically, we used the Classification and Regression Tree (CART) proposed in [73]. Random forests are a type of ensemble learner [74,75] that utilize a collection of decision trees as base learners. Random Forests are widely used for their intepretability, strong dataset generalization abilities, and computational efficiency. We use 100 trees in our model. This number was chosen by analyzing the error of our model as the number of trees was varied (see Figure 4). Our decision trees are grown until the leaf nodes have partitions of, at most, five data points each. The average speed of each leaf node is given by:

(6) $${\bar{s}}_{lb}^{l}=\frac{1}{n}\sum_{i=1}^{n}s_{lb_{i}}^{l}$$

where $s_{lb_{i}}^{l}$ denotes the speed of the ith data point in the node, *n* denotes the number of data points in the node, and ${\bar{s}}_{lb}^{l}$ is the coefficient used to fit a piecewise-constant approximation of the biobot’s speed. Each decision tree is trained using approximately 27% of the training data, obtained via bagging, and 20 of the 60 possible features, chosen randomly. We use the mean squared residual, denoted as $Q_{s}$, as the splitting criterion of our decision trees:

(7) $$Q_{s}=\frac{1}{n}\sum_{i=1}^{n}{\left(s_{lb_{i}}^{l}-{\bar{s}}_{lb}^{l}\right)}^{2}.$$

More information on splitting criteria for decision trees can be found in [74], Chapter 5. The speed of the biobot is determined by traversing each regression tree in the random forest down to a leaf node, obtaining that leaf node’s corresponding ${\bar{s}}_{lb}^{l}$ value, and averaging the results of each of the decision trees as follows:

(8) $${\hat{s}}_{lb}^{l}\left(\tau_{k}\right)=\frac{1}{m}\sum_{j=1}^{m}{\bar{s}}_{lb}^{l,(j)}\left(\tau_{k}\right)$$

where ${\bar{s}}_{lb}^{l,(j)}$ denotes the average speed computed by the jth decision tree, and *m* denotes the number of trees in the random forest (100 in our case). The hyperparameters for $M_{s}$ are shown in Table 2.

#### 4.3.2. Stationarity Detection

Zero-Velocity detection is commonly used in INS applications. It was shown in [61] that the zero-velocity (i.e., zero-speed) state could be accurately tracked in biobots using a random forest model. We used a similar model to construct a stationarity detector $M_{z}:R^{F}\to \left\{0,1\right\}$, where *F* denotes the number of features. The goal of $M_{z}$ is to assign one of two labels to each feature vector, denoting whether the biobot is moving ($M_{z}\left(d\left(\tau_{k}\right)\right)=0$) or stationary ($M_{z}\left(d\left(\tau_{k}\right)\right)=1$). These labels are used in Equation (5) to estimate the biobot’s speed. $M_{z}$ is very similar to $M_{s}$ and the hyperparameters for it are shown in Table 2. The primary differences between $M_{z}$ and $M_{s}$ stem from the fact that $M_{z}$ is a binary classifier. As such, classification decision trees are used and the splitting criterion of the decision trees is different. Specifically, we use the Gini Index, denoted as $Q_{z}$:

(9) $$Q_{z}=\frac{2}{n^{2}}(\sum_{i=1}^{n}I\left(s_{lb_{i}}^{l}=0\right))\cdot (\sum_{i=1}^{n}I\left(s_{lb_{i}}^{l}\neq 0\right))$$

where I(·) denotes the indicator function, $s_{lb_{i}}^{l}$ denotes the speed of the ith data point in the node, and *n* denotes the number of data points in the node. The stationarity of the biobot is predicted by taking the majority vote of the decision trees in $M_{z}$.

Since $d(\tau_{k})$ is associated with a window of data, multiple ground truth video frames could fall within the window. As such, the number of zero-speed video frames needed to flag $d(\tau_{k})$ as stationary is a parameter. We flagged data points as stationary when 100% of their video frames were stationary.

### 4.4. Heading Correction Model

Our AHRS generates estimates of heading for each IMU sample, denoted by ${\tilde{\psi }}_{lb}$; however, these estimates have an error that increases linearly in time, as discussed in Section 4.1. Our heading correction model resolves this issue and has three goals: first, it detrends the error in ${\tilde{\psi }}_{lb}$; secondly, it averages ${\tilde{\psi }}_{lb}$ to produce a heading for each data point, ${\bar{\psi }}_{lb}\left(\tau_{k}\right)$; finally, it corrects ${\bar{\psi }}_{lb}\left(\tau_{k}\right)$ to generate a more accurate estimate of heading of the biobot. Succinctly, this process can be written as:

(10) $${\hat{\psi }}_{lb}\left(\tau_{k}\right)={\bar{\psi }}_{lb}\left(\tau_{k}\right)+M_{\psi }\left(d\left(\tau_{k}\right)\right)$$

where ${\hat{\psi }}_{lb}\left(\tau_{k}\right)$ denotes the final heading estimate that is outputted by the heading correction model and $M_{\psi }:R^{F}\to R$, ${\hat{\Delta }}_{h}\left(\tau_{k}\right):=M_{\psi }\left(d\left(\tau_{k}\right)\right)$, where *F* denotes the number of features. $M_{\psi }$ is a regression model that corrects ${\bar{\psi }}_{lb}\left(\tau_{k}\right)$, where ${\hat{\Delta }}_{h}$ is the estimated heading correction. Equation (10) is solved by splitting the heading correction model into two submodules. The first submodule detrends the AHRS output and averages it to generate ${\bar{\psi }}_{lb}\left(\tau_{k}\right)$; the second submodule is $M_{\psi }$, and it applies the corrective term needed to generate ${\hat{\psi }}_{lb}\left(\tau_{k}\right)$, as mentioned previously. The hyperparameters for $M_{\psi }$ can be found in Table 2.

#### 4.4.1. Detrending the Heading Error

The actual and estimated headings of the biobot are known at the entry and exit times, denoted as $\left[\psi_{lb}\left(t_{s}\right),\psi_{lb}\left(t_{f}\right)\right]$ and $\left[{\tilde{\psi }}_{lb}\left(t_{s}\right),{\tilde{\psi }}_{lb}\left(t_{f}\right)\right]$, where $t_{s}$ and $t_{f}$ denote the entry and exit times, respectively. Using this information, we create a linear model L(t):

(11) $$L\left(t\right)=\left(\frac{E\left(t_{f}\right)-E\left(t_{s}\right)}{t_{f}-t_{s}}\right)\cdot t+E\left(t_{s}\right)$$

where the heading error is defined as, $E\left(\cdot \right)=\psi_{lb}\left(\cdot \right)-{\tilde{\psi }}_{lb}\left(\cdot \right)$. L(t) is then used to detrend the heading error in ${\tilde{\psi }}_{lb}$.

**Figure 5:**
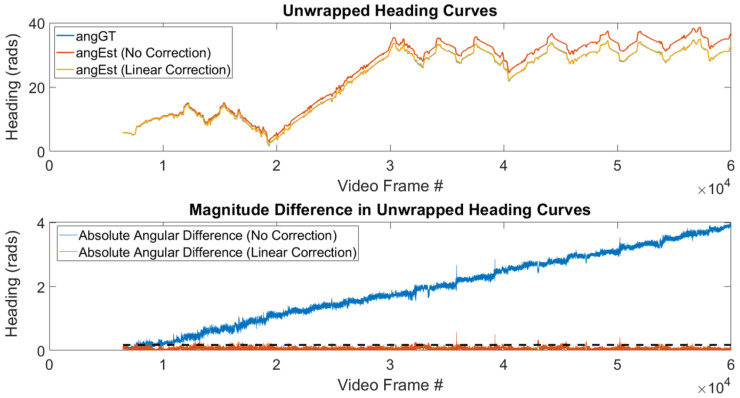
Detrending AHRS Output: The unwrapped heading of the biobot is shown for a 30-min trial. The **top graph** shows the heading in radians and the **bottom graph** shows the original error between the estimated and ground truth headings as well as the error after detrending the AHRS output. The dashed line represents a heading error of $10^{\circ }$. Notice how the error of the detrended AHRS does not grow over time.

The heading associated with the kth feature vector, ${\bar{\psi }}_{lb}\left(\tau_{k}\right)$, is computed by averaging the AHRS output for that window:

(12) $${\bar{\psi }}_{lb}\left(\tau_{k}\right)=\frac{1}{n_{k}}\sum_{i=1}^{n_{k}}\left({\tilde{\psi }}_{lb}\left(t_{i}\right)+L\left(t_{i}\right)\right)$$

where $n_{k}$ denotes the number of IMU samples in the kth data point’s window, and *i* denotes the AHRS output for the ith IMU sample in the window. Figure 5 shows the effect of detrending the heading error on a biobot dataset. In this figure, the detrending algorithm reduces the linear error growth to a constant error that fluctuates due to sensor noise.

#### 4.4.2. Learning Heading Corrections

We designed $M_{\psi }$ to be a random forest of CART regression trees, similar to $M_{s}$. The goal of $M_{\psi }$ is to correct the data point’s heading estimate using $d(\tau_{k})$. This heading correction, denoted as $\Delta_{h}=\psi_{lb}-{\bar{\psi }}_{lb}$, is needed to remove the error that is introduced as a consequence of averaging the original AHRS estimates. $M_{\psi }$ approximates $\Delta_{h}$ using a piecewise-constant function, where the coefficient associated with each leaf node is computed from the average of that particular leaf node’s data points, ${\bar{\Delta }}_{h}=\frac{1}{n}\sum_{i=1}^{n}\Delta_{h_{i}}$. The mean-squared residual is used as the splitting criterion:

(13) $$Q_{\psi }=\frac{1}{n}\sum_{i=1}^{n}{\left(\Delta_{h_{i}}-{\bar{\Delta }}_{h}\right)}^{2}$$

where *i* denotes the ith data point in the node and *n* denotes the number of data points in the node. $\Delta_{h}$ is predicted by averaging the predictions of each of the decision trees in $M_{\psi }$:

(14) $${\hat{\Delta }}_{h}\left(\tau_{k}\right)=\frac{1}{n}\sum_{j=1}^{n}{\bar{\Delta }}_{h_{j}}\left(\tau_{k}\right)$$

where ${\bar{\Delta }}_{h_{j}}$ denotes the average heading correction computed by the jth decision tree, and *n* denotes the number of trees in the random forest (100 in our case). The estimated heading of the biobot at time $\tau_{k}$, ${\hat{\psi }}_{lb}\left(\tau_{k}\right)$, is computed using Equation (10).

### 4.5. Trajectory Estimation

Thus far, we have discussed how to obtain an estimate of the biobot’s heading, ${\hat{\psi }}_{lb}\left(\tau_{k}\right)$, and speed, ${\hat{s}}_{lb}^{l}\left(\tau_{k}\right)$, for each feature vector, $d(\tau_{k})$. In this section, we will discuss how to use these estimates to obtain an estimate of the biobot’s position, ${\hat{r}}_{lb}^{l}$. Furthermore, we will explain how to estimate the biobot’s state trajectory, T(t), which tracks the biobot’s state over the time interval, $t\in [t_{s},t_{f}$], where $t_{s}$ and $t_{f}$ denote the biobot’s entry and exit times, respectively. Before we begin, we need to introduce the terminology needed to describe T.

We define a trajectory segment to be the state trajectory over a time interval $t\in [t_{s_{i}},t_{f_{i}}]$. Until now, we have described a biobot as having a singular entry point and a singular exit point; however, this needn’t be the case. It is possible for a biobot to have multiple entry and exit points over the course of its trajectory—for example, the biobot could repeatedly enter and leave a rubble stack. The ith entry/exit point is used to define the time bounds of the ith trajectory segment, and the trajectory segments are concatenated in a piecewise fashion to obtain the estimate of the biobot’s state trajectory:

(15) $$T\left(t\right):=\left\{\begin{array}{cc} {\hat{x}}_{1}^{l}\left(t\right) & ,\;t\in [t_{s_{1}},t_{f_{1}})\\ \; & \vdots \\ {\hat{x}}_{n-1}^{l}\left(t\right) & ,\;t\in [t_{s_{n-1}},t_{f_{n-1}})\\ {\hat{x}}_{n}^{l}\left(t\right) & ,\;t\in [t_{s_{n}},t_{f_{n}}] \end{array}\right.$$

where *n* denotes the number of entry/exit points, and the subscript of ${\hat{x}}_{i}^{l}\left(t\right)$ is used to emphasize the fact that the estimated state trajectory is only valid for the ith trajectory segment, which is denoted as $T_{i}$. Additionally, we require that the biobot’s state at the entry point of the ith trajectory segment be identical to its state at the exit point of the previous trajectory segment. This means that $t_{s_{i}}=t_{f_{i-1}}$ and $x^{l}\left(t_{s_{i}}\right)=x^{l}\left(t_{f_{i-1}}\right)$. This requirement ensures that T is an approximation of $x^{l}$.

**Figure 6:**
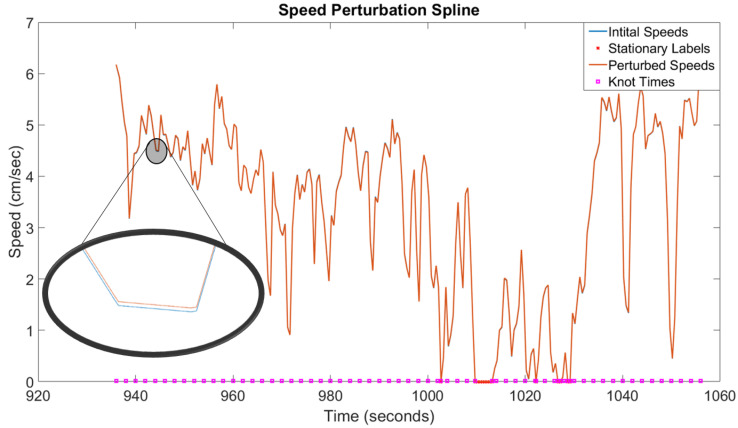
Perturbation Splines: A two-minute section of a biobot’s estimated speed trajectory is shown. The interpolated speed trajectory, ${\hat{s}}_{lb}^{l}\left(t\right)$, is shown in blue. The perturbed speed trajectory, ${\hat{s}}_{lb}^{l*}\left(t\right)$, is shown in orange. ${\hat{s}}_{lb}^{l*}\left(t\right)$ and ${\hat{s}}_{lb}^{l}\left(t\right)$ are magnified in the inset picture to highlight the fact that the trajectories are different. The knot locations of the speed perturbation spline are marked with magenta squares. Additionally, data points that have zero speed are marked with red x’s. Notice how ${\hat{s}}_{lb}^{l*}\left(t\right)$ is zero during the stationary sections of ${\hat{s}}_{lb}^{l}\left(t\right)$—this behavior is achieved by using the algorithm discussed in Section 4.5.3.

The estimated state, ${\hat{x}}_{i}^{l}\left(t\right)$, requires the evaluation of ${\hat{r}}_{{lb}_{i}}^{l}\left(t\right)$, ${\hat{s}}_{{lb}_{i}}^{l}\left(t\right)$, and ${\hat{\psi }}_{{lb}_{i}}\left(t\right)$. We can compute ${\hat{s}}_{{lb}_{i}}^{l}\left(t\right)$ and ${\hat{\psi }}_{{lb}_{i}}\left(t\right)$ by linearly interpolating the speed (Section 4.3) and heading (Section 4.4) estimates obtained from data points that fall within the time bounds of $T_{i}$. In order to satisfy the constraint that ${\hat{r}}_{{lb}_{i}}^{l}\left(t_{f_{i}}\right)=r_{{lb}_{i}}^{l}\left(t_{f_{i}}\right)$, the estimated and actual velocity trajectories need to have the same area under their curves: $\int_{t_{s_{i}}}^{t_{f_{i}}}{\hat{v}}_{{lb}_{i}}^{l}\left(t\right)\;dt=\int_{t_{s_{i}}}^{t_{f_{i}}}v_{{lb}_{i}}^{l}\left(t\right)\;dt$. This is unlikely to happen as it would require the expected error of ${\hat{v}}_{{lb}_{i}}^{l}\left(t\right)$ to be zero—in other words, both ${\hat{s}}_{{lb}_{i}}^{l}$ and ${\hat{\psi }}_{{lb}_{i}}$, the signals that are used to construct ${\hat{v}}_{{lb}_{i}}^{l}\left(t\right)$, would need to have expected errors of zero. To resolve this issue, we perturb the speed and heading trajectories with piecewise-cubic splines (described in Section 4.5.1) so that ${\hat{r}}_{{lb}_{i}}^{l}\left(t_{f_{i}}\right)=r_{{lb}_{i}}^{l}\left(t_{f_{i}}\right)$. The perturbed speed and heading trajectories are denoted as ${\hat{s}}_{{lb}_{i}}^{l*}$ and ${\hat{\psi }}_{{lb}_{i}}^{*}$, respectively, and are generated as follows:

(16) $$\begin{array}{cc} {\hat{s}}_{{lb}_{i}}^{l*}\left(t\right) & ={\hat{s}}_{{lb}_{i}}^{l}\left(t\right)+S_{s_{i}}\left(t\right)\\ {\hat{\psi }}_{{lb}_{i}}^{*}\left(t\right) & ={\hat{\psi }}_{{lb}_{i}}\left(t\right)+S_{\psi_{i}}\left(t\right) \end{array}$$

where $S_{s_{i}}\left(t\right)$ and $S_{\psi_{i}}\left(t\right)$ denote the speed and heading perturbation splines, respectively. The perturbed speed and heading trajectories, illustrated in Figure 6, are then used to compute ${\hat{r}}_{{lb}_{i}}^{l}\left(t\right)$:

(17) $$\begin{array}{cc} {\hat{r}}_{{lb}_{i}}^{l}\left(t\right) & =r_{{lb}_{i}}^{l}\left(t_{s_{i}}\right)+\int_{t_{s_{i}}}^{t}{\hat{v}}_{{lb}_{i}}^{l}\left(t\right)\;dt\\ {\hat{v}}_{{lb}_{i}}^{l}\left(t\right) & ={\left[{\hat{s}}_{{lb}_{i}}^{l*}\left(t\right)\cdot \cos\left({\hat{\psi }}_{{lb}_{i}}^{*}\left(t\right)\right)\;,\;{\hat{s}}_{{lb}_{i}}^{l*}\left(t\right)\cdot \sin\left({\hat{\psi }}_{{lb}_{i}}^{*}\left(t\right)\right)\right]}^{⊤} \end{array}$$

where ${\hat{r}}_{{lb}_{i}}\left(t\right)$ and ${\hat{v}}_{{lb}_{i}}\left(t\right)$ denote the estimated position and velocity trajectories of ${\hat{x}}_{i}^{l}\left(t\right)$, respectively.

To summarize, the biobot’s estimated trajectory, T(t), consists of a set of trajectory segments, ${\left\{{\hat{x}}_{i}^{l}\left(t\right)\right\}}_{1..n}$, where the ith trajectory segment is constructed by using splines to perturb its speed and heading trajectories so that ${\hat{r}}_{{lb}_{i}}^{l}\left(t_{f_{i}}\right)=r_{{lb}_{i}}^{l}\left(t_{f_{i}}\right)$.

#### 4.5.1. Perturbation Spline Construction

$S_{s}\left(t\right)$ and $S_{\psi }\left(t\right)$ are the speed and heading perturbation splines that are needed to correct the speed and heading for a trajectory segment so that the boundary condition, ${\hat{r}}_{lb}^{l}\left(t_{f}\right)=r_{lb}^{l}\left(t_{f}\right)$, is satisfied—this process was described in Equation (16). The index *i* is reused in this section to indicate the ith spline piece of a particular trajectory segment. The speed and heading perturbation splines of a trajectory segment are clamped piecewise-cubic splines that have the following form:

(18) $$S_{i}\left(t\right)=c_{i1}{\left(t_{f_{i}}-t\right)}^{3}+c_{i2}{\left(t-t_{s_{i}}\right)}^{3}+c_{i3}\left(t-t_{s_{i}}\right)+c_{i4}\left(t_{f_{i}}-t\right),\;t\in \left[a_{i},b_{i}\right]$$

where $a_{i}$ denotes the start time of the spline, $b_{i}$ denotes the end time of the spline, and ${\left\{c_{ij}\right\}}_{j=1}^{4}$ are the four coefficients of $S_{i}$. The spline pieces are concatenated to form the entire perturbation spline:

(19) $$S\left(t\right):=\left\{\begin{array}{cc} S_{1}\left(t\right) & ,\;t\in [a_{1},b_{1})\\ \; & \vdots \\ S_{n-1}\left(t\right) & ,\;t\in [a_{n-1},b_{n-1})\\ S_{n}\left(t\right) & ,\;t\in [a_{n},b_{n}] \end{array}\right.$$

where *n* denotes the number of pieces in the spline, $S_{i}$ takes the form described by Equation (18), $a_{1}:=t_{s}$, $b_{n}:=t_{f}$, and $a_{i}=b_{i-1}$ for i=2..n. The duration of each spline piece, $\Delta t:=b_{i}-a_{i}$, is one of the four hyperparameters of the trajectory estimation algorithm, shown in Table 3.

Each of the spline pieces has a set of four coefficients that can be modified to alter the shape of the spline. Since we are interested in using the splines to perturb the speed and heading trajectories, it makes sense to define the coefficients of Equation (18) in terms of the spline piece’s knot locations, denoted as *y*, as these locations will control how much the speed and heading are perturbed at specific times. Each spline piece has two knot locations, obtained by evaluating the spline piece at the start and end times, and denoted as $y_{i}:=\left\{S_{i}\left(a_{i}\right),S_{i}\left(b_{i}\right)\right\}=\left\{y_{i0},y_{i1}\right\}$. Additionally, to ensure that the spline pieces fit together, we will also need to consider the first time derivative of $S_{i}\left(t\right)$:

The first time derivative evaluated at the knot locations is denoted as $m_{i}:=\left\{S_{i}^{\prime }\left(a_{i}\right),S_{i}^{\prime }\left(b_{i}\right)\right\}=\left\{m_{i0},m_{i1}\right\}$. Evaluating $S_{i}\left(t\right)$ and $S_{i}^{\prime }\left(t\right)$ at the knot locations generates the following four Equations:

(20) $$\begin{array}{cc} S_{i}\left(a_{i}\right) & =y_{i0}:=c_{i1}{\left(\Delta t\right)}^{3}+c_{i4}\Delta t \end{array}$$

(21) $$\begin{array}{cc} S_{i}\left(b_{i}\right) & =y_{i1}:=c_{i2}{\left(\Delta t\right)}^{3}+c_{i3}\Delta t \end{array}$$

(22) $$\begin{array}{cc} S_{i}^{\prime }\left(a_{i}\right) & =m_{i0}:=-3c_{i1}{\left(\Delta t\right)}^{2}+c_{i3}-c_{i4} \end{array}$$

(23) $$\begin{array}{cc} S_{i}^{\prime }\left(b_{i}\right) & =m_{i1}:=3c_{i2}{\left(\Delta t\right)}^{2}+c_{i3}-c_{i4} \end{array}$$

where $\Delta t:=b_{i}-a_{i}$. Equations (20)–(23) can be solved to find the coefficients of each spline piece:

(24) $$\begin{array}{cc} c_{i1} & =\frac{c_{i3}-c_{i4}-m_{i0}}{3{\left(\Delta t\right)}^{2}},\\ c_{i2} & =\frac{m_{i1}-c_{i3}+c_{i4}}{3{\left(\Delta t\right)}^{2}},\\ c_{i3} & =\frac{-3y_{i0}+6y_{i1}-\Delta t\left(m_{i0}+2m_{i1}\right)}{3\Delta t},\\ c_{i4} & =\frac{6y_{i0}-3y_{i1}+\Delta t\left(2m_{i0}+m_{i1}\right)}{3\Delta t}. \end{array}$$

Each spline piece has its own set of $y_{i}$ and $m_{i}$ parameters; however, there are two restrictions that limit the values that these parameters can take. The first restriction is that the perturbation spline must be zero at a trajectory segment’s entry and exit points because those states are known and should remain unaltered. The implication of this is that $y_{10}=y_{n1}=0$, where *n* denotes the final spline piece of S(t). The second restriction is that the perturbation spline and its first derivative must be continuous. This necessitates that the following two statements be true: $y_{i0}=y_{i-1,1}$ and $m_{i0}=m_{i-1,1}$. These two restrictions mean that each perturbation spline will have 2n degrees of freedom, where *n* is the number of spline pieces in *S*. As such, each trajectory segment will have 4n optimizable parameters since each trajectory segment contains both a speed perturbation spline and a heading perturbation spline:

(25) $$\frac{dS_{i}\left(t\right)}{dt}=-3c_{i1}{\left(t_{f_{i}}-t\right)}^{2}+3c_{i2}{\left(t-t_{s_{i}}\right)}^{2}+c_{i3}-c_{i4}.$$

#### 4.5.2. Perturbation Spline Optimization

We denote the 4n optimizable parameters of a trajectory segment’s perturbation splines as $\theta =[\theta_{s},\theta_{h}]$, where $\theta_{s}$ are the parameters associated with the speed perturbation spline and $\theta_{h}$ are the parameters associated with the heading perturbation spline.

Recall that the goal of our navigation system is to solve the two-point boundary problem introduced in Equation (2). The cost functional itself, J(θ), measures the distance between the true and estimated state trajectories. The estimated speed and heading trajectories, ${\hat{s}}_{lb}^{l}$ and ${\hat{\psi }}_{lb}$, were constructed by linearly interpolating the estimates obtained via Equations (5) and (10). These estimates represent our best guess of the actual speed and heading trajectories since they are constructed from models trained to minimize the error in the speed and heading estimates, as described in Equations (7), (9), and (13). Additionally, we know that ${\hat{r}}_{lb}^{l}\left(t\right)$ can be constructed by combining ${\hat{s}}_{lb}^{l}$ and ${\hat{\psi }}_{lb}$ as described in Equations (16) and (17). This means that we already have the ${\hat{x}}^{l}\left(t\right)$ that minimizes J(θ), sans the constraints. We also know the biobot’s state at the entry and exit times. This means that we can define ${\hat{s}}_{lb}^{l}\left(t_{s}\right):=s_{lb}^{l}\left(t_{s}\right)$, ${\hat{\psi }}_{lb}\left(t_{s}\right):=\psi_{lb}\left(t_{s}\right)$, ${\hat{s}}_{lb}^{l}\left(t_{f}\right):=s_{lb}^{l}\left(t_{f}\right)$, and ${\hat{\psi }}_{lb}\left(t_{f}\right):=\psi_{lb}\left(t_{f}\right)$ so that all constraints involving speed and heading are satisfied. Additionally, since the biobot’s state is known at the time of entry, we can define ${\hat{r}}_{lb}^{l}\left(t_{s}\right):=r_{lb}^{l}\left(t_{s}\right)$ so that the the starting position constraint is satisfied. As a result of these manipulations, all of the constraints are satisfied except for the end position constraint, ${\hat{r}}_{lb}^{l}\left(t_{f}\right)=r_{lb}^{l}\left(t_{f}\right)$. This endpoint constraint is the reason why we require the speed and heading perturbation splines, and the rest of this section discusses how to optimize these perturbation splines so that the end position constraint is satisfied.

We define a surrogate cost functional for each trajectory segment, denoted as $\tilde{J}$, which aims to minimize the perturbation of our estimates, ${\hat{s}}_{lb}^{l}$ and ${\hat{\psi }}_{lb}$, by placing a weighted cost on the amount of speed and heading perturbation. Additionally, we incorporate the trajectory segment’s end position constraint into $\tilde{J}$ as a weighted penalty term, ensuring that the end position constraint can be satisfied to an ϵ amount. The cost $\tilde{J}$ has the following form:

(26) $$\tilde{J}\left(\theta \right)=\int_{t_{s}}^{t_{f}}\left(W_{s}\cdot S_{s}{\left(t;\theta_{s}\right)}^{2}\;+\;W_{\psi }\cdot S_{\psi }{\left(t;\theta_{h}\right)}^{2}\right)\;dt+W_{r}\cdot ||r_{lb}^{l}\left(t_{f}\right)-{\hat{r}}_{lb}^{l}\left(t_{f};\theta \right)|{|}^{2}.$$

The first term is obtained by numerically integrating the integrand using a sampling rate of 30 Hz. $W_{s}$, $W_{\psi }$, and $W_{r}$ are weights that adjust the impact of the amount of speed perturbation, amount of heading perturbation, and end position constraint violation, respectively. These weights are hyperparameters for the trajectory estimation algorithm and the values that we used can be found in Table 3.

$\tilde{J}\left(\theta \right)$ is optimized using the *fminunc* function of the MATLAB *Optimization* toolbox [76]. This particular function uses the Broyden–Fletcher–Goldfarb–Shanno (BFGS) algorithm ([77], Chapter 6), where the line search ([77], Chapter 3) is performed via a cubic interpolation function. It should be noted that none of the parameters in θ are shared between trajectory segments. This means that each trajectory segment in T can be optimized in parallel, which will increase the algorithm’s computational efficiency.

#### 4.5.3. Handling Stationary Points on the Perturbation Spline

The perturbation splines should not alter the biobot’s speed and heading trajectories when the biobot is stationary. To guarantee this, the perturbation splines must be zero when the biobot is stationary. This can be accomplished by first creating the perturbation spline that is described in Equations (18)–(25). The resulting perturbation spline, S(t), is then modified using the following steps:

1. Stationary points are defined to be points where ${\hat{s}}_{lb}^{l}\left(\tau_{k}\right)=0$. Stationary intervals occur whenever there are two, or more, consecutive stationary points. Find all stationary points and stationary intervals in S(t).
2. Each interval of stationary points will become its own spline piece with coefficients equal to zero: $y_{i}=m_{i}=0$. The new spline piece will start and end at the first and last points of the stationary interval, respectively.
3. Stationary points will become the ends of spline pieces, and the knot location and knot derivative at the stationary points will be zero. This is accomplished by:
  (a) Any spline piece that **falls entirely within** a stationary interval is removed from S(t).
  (b) Any spline piece that **ends** at a stationary point has coefficients: $y_{i1}=m_{i1}=0$.
  (c) Any spline piece that **begins** at a stationary point has coefficients: $y_{i0}=m_{i0}=0$.
  (d) Any spline piece that **contains** stationary intervals and/or stationary points is split into multiple spline pieces such that the new spline pieces terminate on a stationary point:
    i If both ends of a spline piece are stationary points, then the spline piece will take the form described in step 2.
    ii If one end of the spline piece is a stationary point, then it will take the form of 3b or 3c, depending on whether the stationary point is at the beginning or end of the spline piece.

Once S(t) has been altered, the remaining optimizable coefficients can be optimized using Equation (26), as described in Section 4.5.2. A positive side effect of this algorithm is that it can reduce the total number of optimizable parameters in S(t) when the biobot is stationary for prolonged periods of time.

### 4.6. Enhancing the Ground Truth

Our trajectory estimation algorithm (Section 4.5) uses two random forest regression models to estimate a biobot’s speed (Section 4.3) and heading (Section 4.4). These models are only as good as the ground truth data that is used to train them, so it is imperative that we use ground truth speeds and headings that are as accurate as possible. A key component of this is ensuring that the ground truth speeds and headings integrate to match the ground truth positions. We use video recordings to obtain the ground truth state, $x^{l}$, and the specifics of this are detailed in Section 5.4.1. In this section, we will present the algorithm that we use to ensure that the ground truth speeds and headings are correct—that they integrate to match the ground truth positions obtained from the video data.

The ground truth video data give us a discrete set of ground truth speeds and ground truth headings. We linearly interpolate these discrete speeds and headings to generate continuous speed and heading trajectories for the ground truth, denoted as $s_{lb}^{l}$ and $\psi_{lb}$, respectively. We will use the same notation as in Equation (16) to differentiate the original interpolated trajectories from their perturbed counterparts. We compute the ground truth speeds and headings using the same approach that was used for trajectory estimation. First, we split the ground truth state trajectory into a series of trajectory segments, as described in Equation (15), which we shall denote as $T_{G}\left(t\right)$. The starts and ends of the trajectory segments in $T_{G}$ are arbitrary—we use trajectory segments that are one minute each, but this needn’t be the case. Once $T_{G}$ has been created, we define the perturbed ground truth speeds and headings for each trajectory segment as $s_{{lb}_{i}}^{l*}$ and $\psi_{{lb}_{i}}^{*}$, respectively, using a similar form to Equation (16):

(27) $$\begin{array}{cc} s_{{lb}_{i}}^{l*}\left(t\right) & =s_{{lb}_{i}}^{l}\left(t\right)+S_{G_{s_{i}}}\left(t\right)\\ \psi_{{lb}_{i}}^{*}\left(t\right) & =\psi_{{lb}_{i}}\left(t\right)+S_{G_{\psi_{i}}}\left(t\right) \end{array}$$

where the only difference is that we are now using interpolated ground truth speeds and headings as opposed to interpolated estimates of the ground truth speed and heading. The ground truth perturbation splines of the ith trajectory segment, denoted as $S_{G_{s_{i}}}\left(t\right)$ and $S_{G_{\psi_{i}}}\left(t\right)$, are piecewise-cubic perturbation splines that take the same form as the perturbation splines described in Equations (18)–(25). Additionally, the coefficients of $S_{G_{s_{i}}}\left(t\right)$ and $S_{G_{\psi_{i}}}\left(t\right)$ take the form of Equation (24). Finally, we use an almost identical variant of the algorithm defined in Section to ensure that the ground truth perturbation splines do not alter the ground truth speed and heading trajectories when the biobot is stationary. The *only* difference in the algorithm is that the stationary points are determined by using the ground truth speeds obtained from the video frames, denoted as $s_{lb}^{l}$, instead of the speed estimates, ${\hat{s}}_{lb}^{l}$.

Using this information, we define a cost functional for each ground truth trajectory segment, ${\tilde{J}}_{G}$, which has a form similar to Equation (26):

(28) $$\begin{array}{cc} {\tilde{J}}_{G}\left(\theta_{G}\right)= & \int_{t_{s}}^{t_{f}}(W_{G_{r1}}||r_{lb}^{l}\left(t\right)-{\tilde{r}}_{lb}^{l}\left(t;\theta_{G}\right)|{|}^{2}+W_{G_{s}}\cdot S_{G_{s}}{\left(t_{j};\theta_{G,s}\right)}^{2}+W_{G_{\psi }}\cdot S_{G_{\psi }}{\left(t;\theta_{G,h}\right)}^{2})\;dt\\ & \;\;\;\;\;\;\;+W_{G_{r2}}||r_{lb}^{l}\left(t_{f}\right)-{\tilde{r}}_{{lb}_{i}}^{l}\left(t_{f};\theta_{G}\right)|{|}^{2}, \end{array}$$

where ${\tilde{r}}_{{lb}_{i}}^{l}$ is the perturbed ground truth obtained by integrating $s_{lb}^{l*}\left(t\right)$ and $\psi_{lb}^{*}\left(t\right)$. The only difference between Equations (28) and (26) is the introduction of the $||r_{{lb}_{i}}^{l}\left(t\right)-{\tilde{r}}_{{lb}_{i}}^{l}\left(t;\theta_{G}\right)|{|}^{2}$ term, which tracks how much the perturbed ground truth position differs from the original ground truth position that is obtained from linearly interpolating the video frames, $r_{lb}^{l}\left(t\right)$. The end position constraint is retained as a weighted penalty term because the ground truth trajectory, $T_{G}\left(t\right)$, must be continuous. $W_{G_{r1}}$, $W_{G_{s}}$, $W_{G_{\psi }}$, and $W_{G_{r2}}$ are weights that adjust the emphasis that is placed on matching the ground truth position trajectory, amount of speed perturbation, amount of heading perturbation, and end position constraint violation, respectively. These weights are hyperparameters for the ground truth optimization algorithm and the values that we used can be found in Table 4.

## 5. Experimental Setup

This section details the experimental setup that we used to test and verify our algorithm. The hardware and software that we used are listed, and details are provided as to how we constructed our experimental testbed, as well as the biobots themselves. The section concludes with the experimental procedure that we used to collect data, including how we extracted the video ground truth from the video data.

### 5.1. Hardware

**Figure 7:**
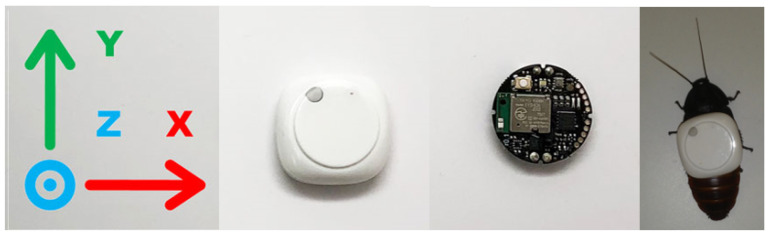
MetaMotion C Sensor Board: From left to right: (1) Coordinate frame of MetaMotion C sensor board (this is the body frame of our algorithm); (2) 3D-printed MetaMotion C case; (3) MetaMotion C PCB; (4) The IMU (inside the case) is mounted to the thorax of the biobot with the +Y direction of the IMU facing in the direction of the biobot’s antennae.

We use a MetaMotion C sensor board (Mbientlab Inc., San Francisco, CA, USA). The MetaMotion C sensor board, shown in Figure 7, is a circular system-on-chip that weighs 3 g and measures 24 mm diameter × 6 mm height. The board has 8 MB of onboard flash storage and is powered by a 3V CR2032 200mAH Lithium coin-cell battery. The board has a 16-bit BMI160 IMU (Bosch GmbH, Reutlingen, Germany) that contains a three-axial accelerometer and a three-axial gyroscope. Processing is handled by a 32-bit Arm Cortex M4F CPU and communication is accomplished via a 2.4 GHz transceiver that uses Bluetooth 4.2 Low Energy. The performance of the BMI160 IMU is shown in Table 5.

We chose the MetaMotion C for two main reasons: first, it is small enough to fit over the thorax of a biobot (see Figure 7), ensuring that it does not alter the biobot’s center of mass; secondly, the MetaMotion C is light enough to not interfere with the biobot’s locomotion. In this study, we just needed to localize the insect; therefore, we did not need the custom backpacks we designed previously for biobotic control of insects (e.g., [17,69,78]).

### 5.2. Arena

**Figure 8:**
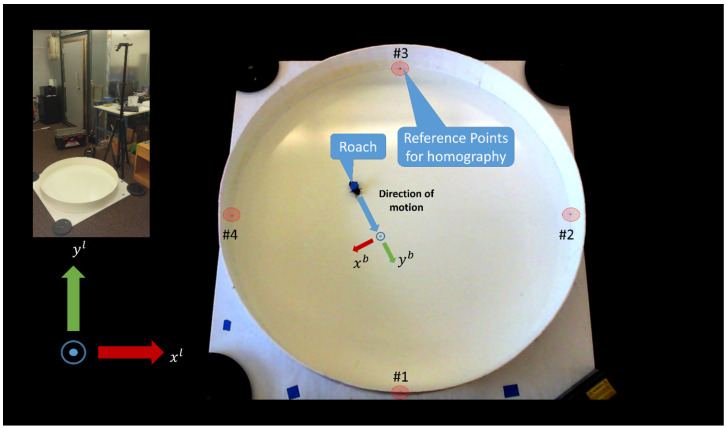
Experimental Setup: Circular arena of 115 cm diameter. Camera and perspective views are presented. The blue object on top of the roach is the IMU. The local tangent reference frame (frame *l*) and roach body frame (frame *b*) are also illustrated. Note that the roach body frame is centered on the IMU and the local tangent reference frame has its origin at the center of the circular arena—these frames have been shifted in this figure for illustration purposes only.

All data were taken inside of a circular arena with a diameter of 115 cm, shown in Figure 8. This circular arena is inscribed inside of a 48” (approx. 122 cm) square of plywood with 155 mm high walls made of poster board. The walls were coated with petroleum jelly to prevent the biobot from climbing on them. Weights were placed at the corners of the plywood base to ensure that the arena did not shift relative to a LifeCam HD-3000 (Microsoft, Redmond, WA, USA) that was mounted 74” (approx. 188 cm) off the ground via a tripod. A laptop was connected to the camera to stream 1280 × 720 resolution video at 30 frames per second.

The reference frame for the system has its origin at the center of the arena, and is aligned with the four points that are numbered in Figure 8. The aforementioned four points were used for computing the homography to convert image space (pixels) to the local tangent reference frame (centimeters), which forms our physical space. The four points form two diameters, and the intersection of these diameters was used to find the center of the circular arena in the physical space. Figure 8 highlights the important components of the arena and includes both a perspective view of the setup and a camera view.

### 5.3. Biobotic Agent

The biobot is a non-instrumented (see Section 5.1) adult female Madagascar hissing cockroach (*Gromphadorhina portentosa*) that measures roughly 60 mm length × 30 mm width (see Figure 7). The roach was taken from a colony that we have raised at NC State since 2013. Additionally, the biobot was kept near room temperature, 75–80 °F, and fed a diet of dog treats.

### 5.4. Data Collection

The MetaMotion C board (Firmware version 1.3.7) was mounted on the thorax of a biobot as shown in Figure 7. The biobot was then placed inside the arena as shown in Figure 8 and allowed to move around inside the arena for 30 min while accelerometer and gyroscope data was logged to the MetaMotion C’s internal storage. The accelerometer and gyroscope ranges were set to ±2 g and ±500 ${}^{\circ }$/s, respectively (see Table 5). These values were chosen because the biobot’s movement never exceeded these sensor limits. Both sensors were sampled at 100 Hz, which is consistent with the IMU sampling rate used in [69]. The IMU was interfaced via Mbientlab’s MetaBase app (version 3.3.0 for Android) on an Android 6.0.1 device.

Video data were used to determine the ground truth position, speed, and heading of the biobot and this is discussed further in Section 5.4.1. The IMU data and video data were synchronized by tapping the IMU three times before the experiment and three times after the experiment. The synchronization taps could be located in both the video feed and the inertial signals, allowing us to map the video time (measured in seconds) to the IMU time (measured in Unix Epoch time). We found that a linear mapping was sufficient to align the IMU and video data to within one video frame. The coefficients of the line were found using a least-squares optimization.

The MetaMotion C sensor board does not start/stop the accelerometer and gyroscope at the same time. As such, there is a slight offset in the timestamps associated with these two sensors. To address this, we manually aligned the accelerometer and gyroscope signals to each other by looking at their IMU times (Unix Epoch Time) and discarding any readings before their first shared sample time and any readings after their last shared sample time. This alignment ensured that the number of accelerometer and gyroscope samples was identical. Furthermore, we used the timestamp of the accelerometer to mark the IMU samples (i.e., the timestamp for the ith accelerometer/gyroscope reading was defined to be the ith accelerometer timestamp). This alignment method allowed us to align the IMU sensors to within two milliseconds of each other. The two millisecond misalignment between the accelerometer and gyroscope was acceptable because our video ground truth was only accurate to one video frame (1/30 s). The protocol for data collection is as follows:

1. Start the MetaBase app and configure the MetaMotion C board to log data internally.
2. Attach the MetaMotion C to the thorax of the biobot.
3. Start the video recording and place the biobot in the arena.
4. Start IMU data logging via the MetaBase app and tap the IMU three times in succession.
5. Let the biobot move in the arena for 30 min.
6. Tap the IMU three times in succession.
7. Stop IMU data logging via the MetaBase app and stop video recording.
8. Retrieve the data from the MetaMotion C via the MetaBase app.

This protocol was used to create a dataset consisting of nine trials. The same biobot was used for each of the nine trials and the biobot was allowed to rest for at least 24 h between trials. By doing this, we ensured that the biobot was fully rested between trials, thereby eliminating any effects that exhaustion could have on the biobot’s movement.

#### 5.4.1. Video Ground Truth

**Figure 9:**
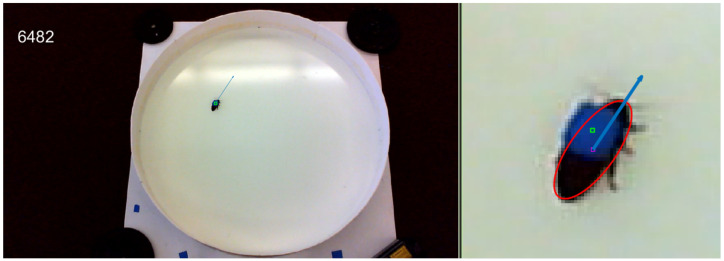
Video Tracker Output: **Left Image**: Biobotic agent inside the arena. The video frame number is shown in the upper left corner of the image. The center of the IMU is marked as a green square and the heading of the biobot is shown with a blue arrow. **Right Image**: Close-up shot of the biobot. The green square denotes the center of the IMU and the blue arrow denotes the biobot’s heading. Additionally, the contour of the biobot’s body is highlighted with a red ellipse and the center of the biobot’s body is marked with a magenta square. All of the aforementioned elements were obtained using the computer vision algorithm described in Section 5.4.1.

The ground truth state of the biobot was obtained from the biobot’s video footage. Specifically, we placed an elliptical bounding box around the biobot and used it to compute the biobot’s position, speed, and heading. To accomplish this, we placed blue tape over the MetaMotion C case (see Figure 9) and used the following approach (summarized in Algorithm 1) to compute the biobot’s pose for each video frame:

1. Compute a background image for the video that excludes the biobot and convert the image to the HSV color space.
2. For each video frame:
  (a) Isolate the elements of the video frame that differ from the background: (i) Convert the video frame to the HSV color space and subtract the background image from the video frame. This generates a difference image. (ii) Define a set of thresholds applied to the S and V channels of the difference image to identify the parts of the image that differ substantially from the background image. As such, the only nonzero pixels in the thresholded difference image will be those of the MetaMotion C and the biobot.
  (b) Find the Meta Motion C by applying a color threshold to the HSV image. In our setup, we applied a color threshold to isolate blue-colored objects since this was the color of the MetaMotion in the video feed (see Figure 9).
  (c) Generate an edge image by applying a Canny edge detector [79] to binary detections of the MetaMotion C and the biobot. Set the pixel locations of the MetaMotion C in the edge image to be zero. The only non-zero pixels in the edge image will belong to the body of the biobot.
  (d) Fit an ellipse to the elliptical edge (i.e., body of the biobot) in the edge image and store the ellipse’s center and orientation in pixel coordinates. This ellipse is the elliptical bounding box that will be used to compute the ground truth state for the video frame.

**Algorithm 1** Pose Algorithm.
**Input:** video *F* thresholds used to detect agent *t_ag_* thresholds used to detect MetaMotion *t_mm_*

**Output:**P, the set of biobot poses

  1:  $b_{hsv}$←C_REATE_B_ACKGROUND_I_MAGE_(F,’hsv’)

  2:  **for each** video frame f∈F **do**

  3:  $f_{hsv}$←C_ONVER_I_MAGE_(*f*,’hsv’)

  4:  $d_{hsv}$←R_EMOVE_B_ACKGROUND_($f_{hsv}$,$b_{hsv}$)

  5:

  6:  $m_{ag}$←F_IND_A_GENT_($d_{hsv}$,$t_{ag})$

  7:  $m_{mm}$←F_IND_M_ETA_M_OTION_($d_{hsv}$,$f_{hsv}$,$t_{mm}$)

  8:

  9:  $e_{ag}$←G_ENERATE_E_DGE_I_MAGE_($m_{ag}$,’C_ANNY_’)

  10:   $e_{bb}$←R_EMOVE_M_ETA_M_OTION_($e_{ag}$,$m_{mm}$)

  11:

  12:   *L*←F_IND_E_LLIPSE_($e_{bb}$)

  13:   $\left[r_{lb}^{l},\psi_{lb}\right]$←E_XTRACT_P_OSE_(*L*)

  14:   Store pose in P

  15: **end for** P

  16: **return** P


We applied a homography to convert the biobot’s elliptical bounding box from image space (pixel coordinates) to physical space (centimeter coordinates)—the reference points used in the homography are shown in Figure 8. The biobot’s heading, $\psi_{lb}$, was defined to be the direction of the major axis of the elliptical bounding box. The biobot’s position, $r_{lb}^{l}$, was defined to be the center of the elliptical bounding box. The speed was determined by computing the biobot’s position displacement over time. Additionally, we used a speed threshold to determine when the biobot was stationary. This was necessary because the biobot’s speed could fluctuate over time when it was stationary due to pixel differences in the biobot’s position between video frames.

The procedure for computing the ground truth does not guarantee that the biobot’s ground truth speed and heading trajectories will integrate to match the biobot’s ground truth position trajectory. We resolved this by using the algorithm discussed in Section 4.6 to refine our ground truth.

## 6. Results and Discussion

We analyzed our navigation framework using a dataset that consists of nine trials. Each trial is approximately 30 min and the trials were conducted using the procedure described in Section 5. The first four trials were used for training and the remaining five trials were used for testing. The hyperparameters for the speed and heading correction models were set to the values specified in Table 2. The hyperparameters for the trajectory estimation algorithm can be found in Table 3 for varying trajectory segment lengths. Ground truth refinement was performed using the hyperparameter values in Table 4 and trajectory segments of one-minute length. All other results were obtained using trajectory segments of two-minute length, unless explicitly stated otherwise. Finally, data points were obtained by using a one-second sliding window with 50% overlap.

### 6.1. Speed Regression

**Figure 10:**
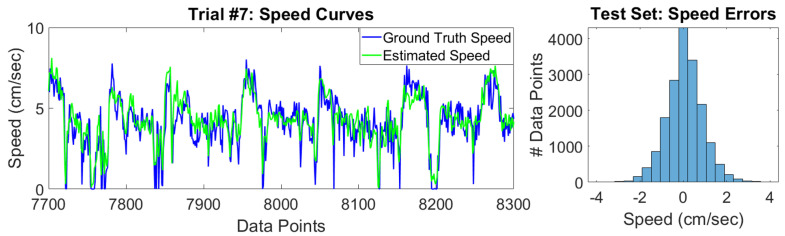
Test Set Speed Errors: (**Left Plot**) A five-minute section of Trial #7’s speed curve is shown, where the estimated and ground truth speeds are compared. (**Right Plot**) Distribution of the speed errors between the estimated and ground truth speeds. Each bin has a width of 0.4 cm/s.

The performance of our speed estimation model is shown in Table 6. Figure 10 highlights a section of trial #7’s estimated speed curve as well as the overall distribution of speed errors in the test set. As expected, the training set error is lower than the test set error because the random forest model was trained to minimize the errors in the training data.

**Figure 11:**
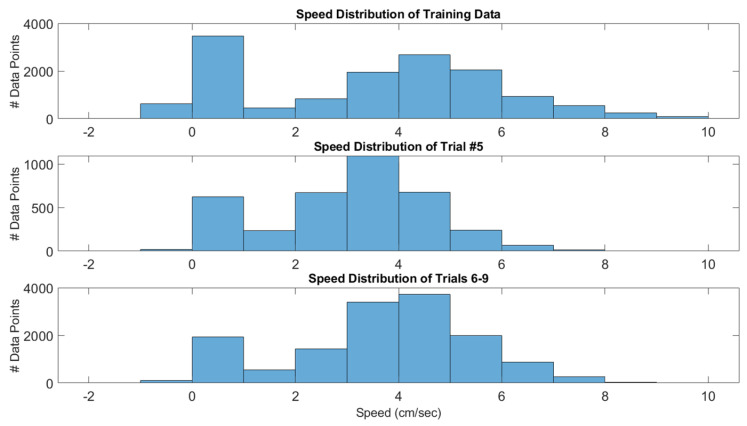
Ground Truth Speed Distributions: Each bin has a width of 1 cm/s. Note that the negative speeds are a result of the perturbation that occurs in the ground truth refinement algorithm. (**Top Plot**) Speed distribution of the training data (trials 1–4). (**Middle Plot**) Speed distribution of trial #5. Trial #5 has a large number of data points in the 2–4 cm/s range, which isn’t well-sampled in the training data; this has an adverse effect on its estimated speed. (**Bottom Plot**) Speed distribution of trials 6–9.

Overall, our model is able to track the ground truth speeds, with an RMSE of less than 1 cm/s for each of the trials (see Table 6). With that said, our model underestimates speeds that have large magnitude. The reason for this is the lack of training samples that have large speeds, as shown in Figure 11. Interestingly, our model has a very low mean signed error, which means that underestimates in the speed are counteracted by overestimates in other data points. The implication of this is that the estimated and true trajectories have comparable lengths.

Lastly, we would like to highlight trial #5 in Table 6, which has a mean signed error that is much larger than the other trials. The reason for this is that trial #5 is abnormally slow, as shown in Figure 11. Specifically, it has a large number of speeds that are 2–4 cm/s, which incidentally happens to be a speed range that is not largely sampled in the training set. This highlights the limitation of the machine learning approach to inertial navigation that was mentioned in Section 2, namely that machine learning approaches depend on having test set data that is similar to the training set data. If we had added trial #5 to the training data, we could have improved the performance of the test set data, especially trial #8, which has a similar speed distribution to trial #5.

### 6.2. Stationarity Detection

The confusion matrix for our stationarity detector can be found in Table 7, where “Stationary” is the positive class and “Moving” is the negative class. Additionally, several performance metrics are shown in Table 8.

The performance metrics show that our stationarity detector has a high accuracy rate; however, this is misleading because the biobot is much more likely to be moving than stationary, as shown in the confusion matrix. As a result, the Matthews Correlation Coefficient (MCC), which shows the correlation between the predicted and true outputs, is a better indicator of the stationarity detector’s performance. Since the MCC is high, we can conclude that the stationarity detector is working. Furthermore, the false-positives and false-negatives are the results of ambiguities and choices in our annotation, which is discussed next.

**Figure 12:**
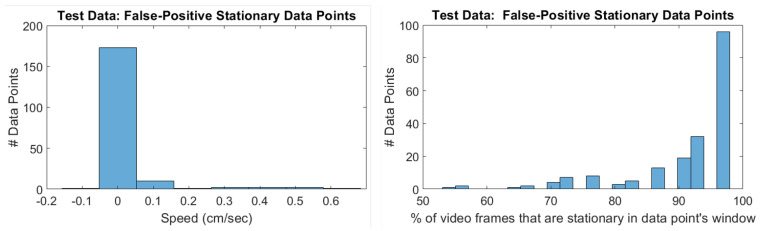
Speed Distribution of False-Positive Stationary Samples: (**Left Plot**) Ground truth speeds for samples falsely flagged as stationary. Bin width of 0.1 cm/s. (**Right Plot**) Percentage of video frames that are labeled as stationary in false positive data points. Bin width of 2%. Many false-positives are caused by data points that have most, but not all, of their video frames flagged as stationary—altering the video frame threshold in the stationarity detector would resolve this issue.

Each data point’s label is created from the video frames that fall within its window, as stated in Section 4.3.2. As a result, the percentage of video frames needed to flag a data point as stationary is a hyperparameter. We set this percentage to be 100% (i.e., *all* of the video frames need to be stationary). Figure 12 shows two things: first, the overwhelming majority of the false-positive data points have zero-speed; secondly, most of the false-positive data points have at least 96% of their video frames as stationary. Since we are using data points that are one second long, with a video frame rate of 30 fps, this means that most of the false-positive data points have 28/30 video frames as stationary. Dropping the number of video frames needed to flag a data point as stationary to 28, instead of 30, would remove most of the false-positive samples. Trial #9, in particular, had a large number of false-positive data points, so making this change would increase its performance.

**Figure 13:**
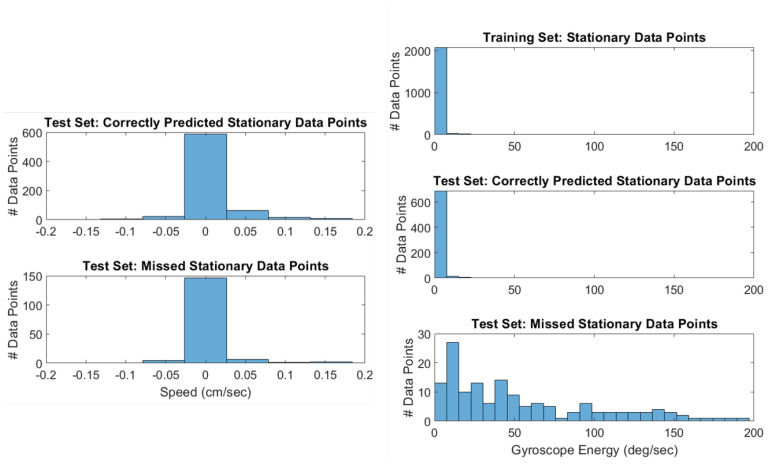
Missed Stationary Samples: (**Top Left Plot**) Ground truth speeds for samples falsely flagged as moving. Bins have widths of 0.04 cm/s. (**Bottom Left Plot**) Ground truth speeds for samples falsely flagged as moving. (**Top Right Plot**) Gyroscope energy in stationary samples for training data. Bins have width of approximately 7.6 deg/s. (**Middle Right Plot**) Gyroscope energy in correctly predicted stationary samples. (**Bottom Right Plot**) Gyroscope energy in missed stationary samples. This shows that false-negatives occur when the biobot is rotating in place (i.e., high gyroscope energy).

Our stationarity detector only considers when an agent has zero-speed. As a consequence, when the biobot is rotating in place, the biobot is still flagged as “stationary”. This is the cause of most of the false-negative data points, as shown in Figure 13. Figure 13 reveals that the overwhelming majority of false-negatives are indeed zero-speed. Furthermore, the speed distribution of the false-negative data points is similar to the speed distribution of the true-positive data points. Figure 13 also shows us that the reason why false-negatives occur is because the biobot is rotating in place, as evidenced by the gyroscope energy. A solution to this issue would be to separate the current stationary label into two labels: “stationary” and “rotating in place”. This would resolve most of the false negatives, and would be especially useful in trials 6–7, which have a large number of instances where the biobot is rotating in place (i.e., false-negatives).

### 6.3. Ground Truth Refinement

**Figure 14:**
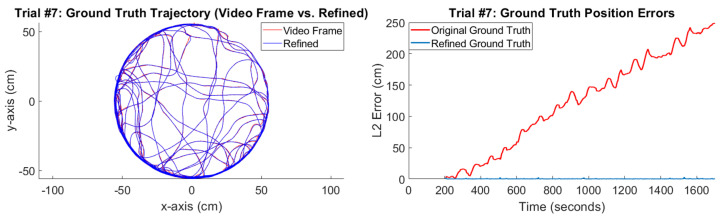
Ground Truth Refinement: (**Left Plot**) Refined Ground Truth Trajectory. Red Line: The position trajectory obtained from the video frames themselves. Blue Line: The position trajectory obtained by integrating the speeds and headings that have been refined via the ground truth refinement algorithm (Section 4.6). (**Right Plot**) Ground Truth Position Errors. The figure shows the distance between the position trajectory obtained from the video frames and the position trajectory that is integrated from the speeds and headings. Red Line: The original speeds and headings that are obtained from the video frames themselves. Blue Line: The refined speeds and headings that are obtained from the ground truth refinement algorithm. The ground truth refinement algorithm corrects the speeds and headings obtained from the video frames so that the position error does not grow over time.

The ground truth refinement algorithm, discussed in Section 4.6, is an optimization algorithm that perturbs the speeds and headings obtained from each video frame (Section 5.4.1) such that they integrate to match the positions in those video frames. Table 9 and Figure 14 show the results of the ground truth refinement algorithm. Table 9 shows the $L^{2}$ error—that is, the distance between the true position trajectory that is obtained from the video frames and the position trajectory that is integrated from the speeds and headings—where the “baseline” method refers to the speeds and headings that are obtained from the video frames themselves, and the “refined” method refers to the perturbed speeds and headings that are obtained from the ground truth refinement algorithm. We see that the ground truth refinement algorithm is required because the position trajectory obtained from the baseline method does not track the true position trajectory. This error occurs because the speed and heading of each video frame is slightly off from the true speed and heading. Furthermore, since this error is uncorrected, it causes the position error to grow linearly over time, as shown in Figure 14. By contrast, the refined speeds and headings obtained from the ground truth refinement algorithm are perturbed so that the position error does not grow over time. Figure 14 also shows what the ground truth trajectory looks like after refinement.

### 6.4. Heading Correction

**Figure 15:**
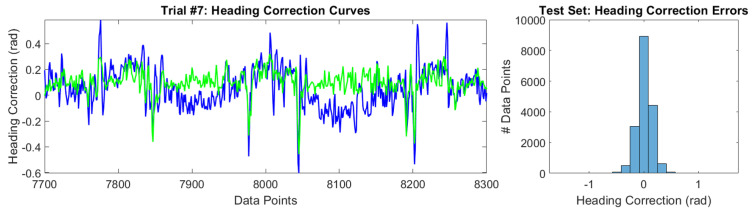
Test Set Heading Correction Errors: (**Left Plot**) A five-minute section of Trial #7’s heading correction curve is shown, where the estimated heading correction (Green line) is compared against the ground truth heading correction (Blue line). (**Right Plot**) Distribution of the heading correction errors between the estimated and ground truth heading corrections. Each bin has a width of 0.17 radians.

The heading correction model is broken into two components, as discussed in Section 4.4: the first component detrends the error in the AHRS output, and its results were shown in Figure 5; the second component is a regression model that estimates the heading correction needed to align the AHRS output with the refined ground truth headings. The second component is what is presented in this section and the performance of the heading correction regression model is shown in Table 10. Additionally, a sample of a heading correction curve is shown in Figure 15 where the distribution of the heading correction errors are also displayed. Table 10 reveals that our heading regression model reduced the heading error in 58.24% of the data points in the test set. Furthermore, the heading regression model reduced the average heading error of the test set by 16.65%, where the percentage improvement over using only the detrended AHRS outputs was calculated as follows:

(29) $$\%\;Improvement=\left(1-\frac{Avg.\;Heading\;Error\;with\;Heading\;Corrections}{Avg.\;Heading\;Error\;without\;Heading\;Corrections}\right)\times 100$$

The underlying premise of the heading correction regression model is that heading corrections are independent of the heading trajectory itself; instead, they are determined by the egomotion of the biobot. As evidence in support of this hypothesis, we observed that the distribution of the heading corrections is almost identical between the training and test datasets, even though these datasets comprise nine different heading trajectories.

Furthermore, the similarity between the heading corrections in the training and test datasets explains the consistency in the performance of the heading correction regression model, as shown in Table 10. The only outlier is trial #6, which has a larger error than the other trials because the biobot attempted to climb on the arena wall during the trial.

Lastly, we want to highlight the fact that the mean signed heading correction error is very close to zero for the trials. The implication of this is that overestimates in the heading correction are counteracted by underestimates in other data points, similar to what happened with the speed regression model. As such, the estimated heading trajectory will end near the true heading, even before the estimated heading trajectory is perturbed via the heading perturbation spline.

### 6.5. Trajectory Estimation

The purpose of the trajectory estimation algorithm is to reconstruct the biobot’s state trajectory over a period of time. We have already reported the results pertaining to the speed and heading states, so we will focus on the position trajectory in this section. There are several ways to incorporate the heading correction and speed estimation models, so we will analyze the trajectory estimation algorithm’s performance for these different configurations, which are as follows:

- Configuration $C_{0}$: The baseline configuration. This configuration uses the detrended AHRS outputs, but includes neither the heading correction regression model nor the stationarity detection component of the speed estimation model.
- Configuration $C_{\psi }$: This configuration includes the full heading correction model (i.e., detrended AHRS outputs and heading correction regression model) but does not include the stationarity detector component of the speed estimation model.
- Configuration $C_{\psi ,z_{ideal}}$: This configuration includes the full heading correction model and the **ground truth** stationarity labels—in other words, the stationarity detector is assumed to be an ideal classifier.
- Configuration $C_{\psi ,z}$: This configuration includes the full heading correction model and **estimated** stationarity labels.

Table 11 shows the results of the trajectory estimation algorithm for the four configurations under the following conditions: (1) using ground truth (GT) speeds and GT headings obtained from the GT refinement algorithm; (2) using GT speeds and headings that are estimated from the heading correction model; (3) using estimated speeds and GT headings; and (4) using estimated speeds and headings.

Note that, when using a GT speed or GT heading, we replace any estimate coming from the AHRS or after applying a correction with the corresponding GT signal. In particular, the $C_{0}$ and $C_{\psi }$ configurations are identical when using the GT heading. Table 11 shows that configuration $C_{0}$ is the worst performing configuration when using estimated headings; however, the table also reveals that stationarity detection offers no performance improvement. With that said, stationarity detection provides the ability to reduce the number of optimizable coefficients in the trajectory estimation algorithm. Therefore, an efficient use of the stationarity detector would be to restrict its usage to areas where the biobot is stationary for prolonged periods of time. Furthermore, the average position error of the test set in configuration $C_{\psi }$ and $C_{\psi ,z}$ when using the estimated speeds and headings (“Est. Speed/Est. Heading” column) is 10.31% lower than the position error obtained using configuration $C_{0}$. These two points illustrate the benefit of incorporating the heading correction regression model.

**Figure 16:**
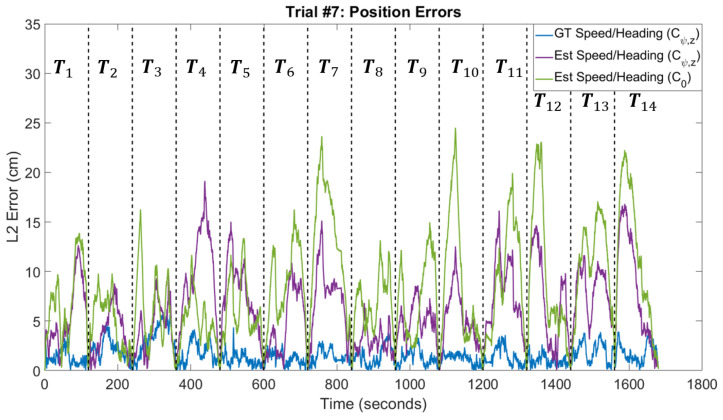
Trial #7 Position Errors: Distance between the ground truth and estimated position trajectories for varying algorithm configurations. $T_{i}$ denotes the ith trajectory segment. **Configuration C_0_:** Detrended AHRS output, no heading correction regression model, and no stationarity detection. **Configuration $C_{\psi ,z}$:** Full heading correction model and estimated stationary labels.

**Figure 17:**
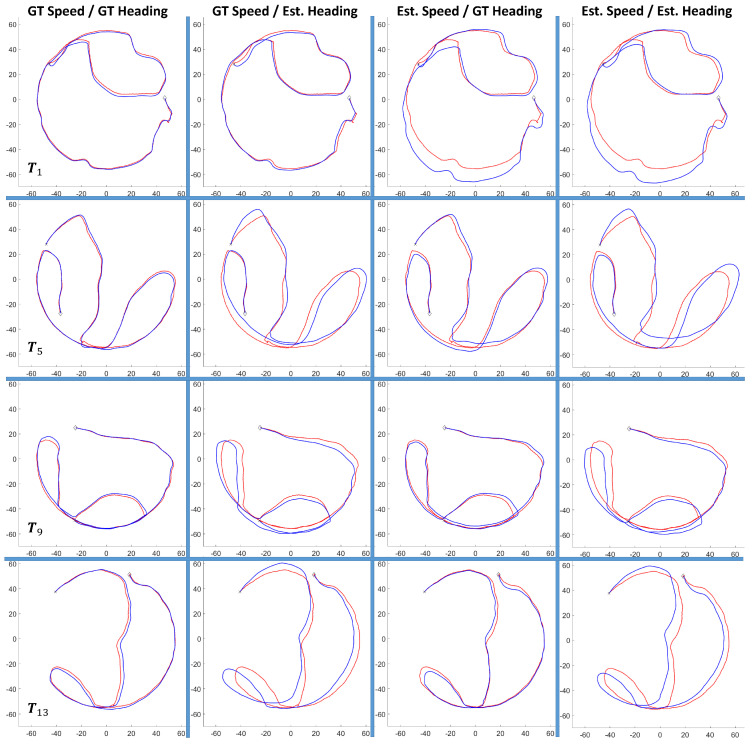
Estimated Trajectory Segments for Trial #7: Estimated position trajectories under varying conditions for Configuration $C_{\psi ,z}$. Each row is a two-minute trajectory segment and each column is a condition. The start point of the trajectory segment is denoted by a black ’x’ and the end point of the trajectory segment is denoted by a black diamond. The horizontal and vertical axes are in centimeters. **Red Line:** Ground truth trajectory. **Blue Line:** Estimated trajectory.

The position errors for trial #7 are shown in Figure 16 for each of the trajectory segments. In this figure, we see that $C_{0}$ has noticeably worse performance for most of the trajectory segments, once again illustrating the importance of the heading correction regression model. It is noteworthy that $C_{0}$ outperforms the other configurations in trajectory segment #4. This occurs because almost all of the heading corrections in trajectory segment #4 are overestimates. This issue could be resolved by improving the quality of the heading correction regression model. Estimated position trajectories for trial #7 are shown in Figure 17. This figure provides an illustration of how the position trajectories look for various two-minute trajectory segments. Appendix A provides more numerical details on the analysis for Trial #7.

Table 12 shows the results of the trajectory estimation algorithm as the trajectory segment length is varied from 2 min to 28 min. This table reveals that the position error increases as the trajectory segments get longer; however, this is to be expected as longer trajectory segments mean that there are less ground truth states that can be used to correct the biobot’s estimated state. It is interesting to note that trial #8 has less average error for 14-min trajectory segments than it does for 7-min trajectory segments. This occurs because trial #8 has an abnormally high heading error in the 22–26 min range, which happens to encompass most of the corresponding 7-min trajectory segment (21–28 min range). This 7-min trajectory segment has 57.1% of its data points taken from this time range, making it more susceptible to the erroneous headings than the 14-min trajectory segment, which only has 28.5% of its data points taken from this window. The other peculiarity in Table 12 comes from trial #6, which has abnormally large position errors. These errors are caused by the biobot attempting to climb on the wall, as discussed in Section 6.4. When this event occurred, the assumption that we made on the direction of gravity in the body frame (see Section 3) became invalid, causing the AHRS readings to become erroneous. If the trajectory segments are short enough (e.g., 2-min trajectory segments), then these erroneous headings are corrected with a subsequent ground truth state before the position error grows too large. If, however, the trajectory segments are too long, then the erroneous AHRS outputs will cause a large position error, hence the abnormally large position errors for trial #6 in Table 12 for trajectory segments that are longer than two minutes.

We analyzed the runtime of our navigation system due to the time-sensitive nature of urban search and rescue. The results are shown in Table 13 for Trial #7 and were obtained in MATLAB 2018b using a desktop computer with the following specifications: Intel Core i7-7700k CPU, Nvidia GeForce GTX Titan X GPU, 64GB RAM, and 64-bit Ubuntu 16.04.6 LTS operating system. We found that our entire algorithm is capable of being executed—from feature extraction to trajectory estimation—in less than two minutes for two-minute trajectory segments. Additionally, since each trajectory segment is parallelizable, it is possible to estimate the entire trial in less than two minutes, assuming that a machine is used that can handle the parallelization without a degradation in performance. Our results were recorded in a video that can be found online at the following URL (https://youtu.be/pgcds0RNqas).

### 6.6. Discussion

We mentioned in Section 6.2 that it may be advantageous to alter the definition of stationarity to only include data points that have both zero-speed and zero-rotation. Making this distinction could allow us to identify data points where the biobot is rotating in place, which would make it possible to prevent speed perturbations while allowing heading perturbations. This would remove situations where the agent moves in a circular arc rather than rotating in place—an example of this behavior can be seen at the start of the $T_{1}$ trajectories in Figure 17.

Figure 16 shows us that the trajectory estimation algorithm can exhibit large position errors towards the center of the trajectory segments. Since this also occurs when ground truth speeds and headings are used, it cannot be explained away by inaccuracies in the regression models. One explanation is that our perturbation splines do not have a sufficient degree of freedom to track the speed and heading trajectories that are far away from the end points. Evidence for this comes from the fact that our cubic splines have four degrees of freedom, and all four of those degrees of freedom are used to satisfy the constraints needed for continuity of the derivatives. As such, each spline piece has coefficients that affect every subsequent spline piece—this can be seen by deriving the $\frac{\partial S_{j}}{\partial m_{i}}$ and $\frac{\partial S_{j}}{\partial y_{i}}$ terms using Equation (18) and Equations (20)–(24). This issue could be resolved by using a quartic spline. The extra degree of freedom would give each spline piece more flexibility to track the speeds and headings. Furthermore, if quartic Bézier curves are used as spline pieces, then continuity of the derivatives can be obtained using only the first two and last two control points. This limits the effect of a spline piece’s coefficients to its immediate successor. Additionally, each spline piece will have one free control point that will not affect any subsequent spline pieces; this control point is free to take any value, altering the shape of the spline piece while retaining the spline’s continuity of the derivatives.

The generalization capabilities of our speed and heading correction regression models are dependent on the data that they are trained on. As we have seen with the speed regression of trial #5, sparsity in the training data can lead to underestimates and/or overestimates in the regression models. This problem can be mitigated by increasing the variety of the speeds and heading corrections in the training data. Additionally, it may be possible to improve the performance of our regression models by incorporating spectral and/or wavelet features, such as the features presented in [61]. Finally, we do not attempt to denoise the IMU signals before sending them through our system. This was sufficient for our data; however, using denoised IMU signals may improve the performance of our models.

As a last point, we’d like to emphasize that our results, while promising, were obtained from a flat circular arena that was devoid of obstacles and other agents. This setup was sufficient to illustrate the principles of our navigation system; however, additional work is required to determine the efficacy of our algorithm in complex environments that are more reminiscent of disaster scenarios.

## 7. Conclusions and Future Work

In this paper, we presented a machine-learning framework for performing inertial navigation using low-cost IMUs. The algorithm posed the navigation problem as a two-point boundary value problem where the goal was to reconstruct an agent’s state trajectory between the start and end points. This was accomplished through the use of models that were capable of estimating an agent’s speed and heading. These speed and heading estimates were then perturbed so that the estimated state trajectory satisfied the boundary conditions. The navigation framework was tested using a biobotic agent in a 2D homogeneous environment. We believe that this new framework would provide the missing localization capability for insect biobots, which is essential for their potential use in USAR applications in the future.

Our algorithm is restricted to 2D environments because we have not implemented a method to determine the direction of gravity that is needed to run the AHRS. As such, an extension to the algorithm would be to design a method (e.g., a regression model) for determining the direction of gravity in the body frame of the IMU, thus allowing the INS to operate in 3D environments. Another extension would be to incorporate the sensors on the biobot that allow it to detect other biobotic agents. These sensors could be used to reduce the error in a biobot’s estimated trajectory by incorporating information about its proximity to other agents. A final extension could be the incorporation of a terrain detector for detecting different types of terrains. Such a system could be of use in non-homogeneous environments, since it would allow our INS to detect the type of terrain and switch to the appropriate regression model(s). Alternatively, the training data could be extended to include varying terrain types. We plan to pursue these extensions in future work.

## Figures and Tables

**Table 1:** Feature vector.

Feature Name	# Features
Mean	6
Variance	6
Skewness	6
Kurtosis	6
Cross-Correlation between Sensors	15
Range (Max Value–Min Value)	6
Mean Absolute Deviation	6
Interquartile Range	6
Gyroscope Energy	3

**Table 2:** Random forest model hyperparameters.

Parameter Names	$M_{s}$	$M_{z}$	$M_{\psi }$
Model Type	Regression	Classification	Regression
Tree Type	CART	CART	CART
Regression Function	Piecewise-Constant	N/A	Piecewise-Constant
Splitting Criterion	Mean-Squared Residual	Gini Index	Mean-Squared Residual
# Trees	100	100	100
% Training Data per Tree	≈ 27%	≈ 5%	≈ 31%
# Features per Tree	20	8	20
# Data Points per Leaf Node	5	1	5
% GT samples to flag $d(\tau_{k})$ as stationary	N/A	100%	N/A

**Table 3:** Trajectory estimation hyperparameters for varying trajectory segment lengths.

Name	Description	2-min. $T_{i}$	7-min. $T_{i}$	14-min. $T_{i}$	28-min. $T_{i}$
Δt	Duration of the perturbation spline pieces (in seconds).	2	7	14	28
$W_{s}$	Weight on the cost associated with speed perturbation.	1	1	1	1
$W_{\psi }$	Weight on the cost associated with heading perturbation.	1	1	1	1
$W_{r}$	Weight on the cost associated with end point, ${\hat{r}}_{lb}^{l}\left(t_{f}\right)$.	120	420	840	1680

**Table 4:** Hyperparameters for Ground Truth Optimization.

Name	Description	Value
Δt	Duration of the perturbation spline pieces (in seconds).	2
$W_{G_{r1}}$	Weight on cost associated with matching $r_{lb}^{l}\left(t\right)$	1
$W_{G_{s}}$	Weight on the cost associated with speed perturbation.	1
$W_{G_{\psi }}$	Weight on the cost associated with heading perturbation.	1
$W_{G_{r2}}$	Weight on the cost associated with end point, ${\hat{r}}_{lb}^{l}\left(t_{f}\right)$.	60

**Table 5:** BMI160 specifications and performance *.

Name	Accelerometer	Gyroscope
Range	±2 g	±500${}^{{}^{\circ}}/$s
Sensitivity	16384 LSB/g	65.6 LSB/${}^{{}^{\circ}}/$s
Sampling Rate	100 Hz	100 Hz
Sensor Noise ($\sqrt{\mathrm{PSD}}$)	180 $\mu g/\sqrt{\mathrm{Hz}}$	$0.007^{{}^{\circ}}/s/\sqrt{\mathrm{Hz}}$
Sensor Noise @100Hz (RMS)	≈1.3 mg	≈0.05${}^{{}^{\circ}}/$s
Sensor Bias (@25 °C)	±40 mg	± 3${}^{{}^{\circ}}/$s
Sensor Bias Temperature Drift	±1 mg/K	0.05${}^{{}^{\circ}}$/s/K
Sensor Sensitivity Temperature Drift	±0.03%/K	±0.02%/K

* All quantities were obtained from the BMI160 datasheet.

**Table 6:** Performance of speed estimation model.

Trial	RMSE (cm/s)	Mean Signed Error (cm/s)
Trial #5	0.7719	0.3219
Trial #6	0.8101	0.0961
Trial #7	0.8711	0.0122
Trial #8	0.8929	−0.1040
Trial #9	0.8411	0.0720
Test Data (Trials 5–9)	0.8387	0.0794
Training Data (Trials 1–4)	0.4034	0.003

**Table 7:** Test set confusion matrix for stationarity detection.

		Predicted	
		Stationary	Moving
True	Stationary	708	162
	Moving	193	16887

**Table 8:** Performance of stationarity detector.

Trial	Accuracy (%)	Precision (%)	Recall (%)	F_1_ Score	MCC
Trial #5	97.32	77.78	86.82	0.8205	0.8075
Trial #6	98.86	87.37	74.77	0.8058	0.8026
Trial #7	98.72	72.41	74.12	0.7326	0.7260
Trial #8	97.45	86.72	80.20	0.8333	0.8203
Trial #9	97.80	64.38	83.74	0.7279	0.7234
Test Data (Trials 5–9)	98.02	78.58	81.38	0.7995	0.7893
Training Data (Trials 1–4)	100	∼	∼	∼	∼

**Table 9:** $L^{2}$ Error (cm) for ground truth positions.

Trial	Baseline: Mean Error (Error Std. Dev.)	Refined: Mean Error (Error Std. Dev.)
Trial #1	35.34 (20.87)	0.255 (0.332)
Trial #2	184.05 (136.61)	0.269 (0.281)
Trial #3	37.97 (40.59)	0.214 (0.256)
Trial #4	60.51 (61.32)	0.235 (0.261)
Trial #5	69.68 (55.51)	0.253 (0.380)
Trial #6	59.08 (21.25)	0.278 (0.464)
Trial #7	146.94 (84.72)	0.241 (0.261)
Trial #8	74.82 (44.09)	0.273 (0.281)
Trial #9	87.29 (43.33)	0.262 (0.313)
**Average**	83.96 (56.48)	0.253 (0.314)

**Table 10:** Performance of heading correction model.

Trial	RMSE	Mean Signed	% of Data Points	Trial’s Heading
#	(rad)	Error (rad)	with Improved Heading	Improvement (%)
Trial #5	0.1363	0.0122	57.67	17.25
Trial #6	0.4846	0.0692	50.14	3.82
Trial #7	0.1451	−0.0052	65.16	27.54
Trial #8	0.1341	−0.0140	66.63	29.12
Trial #9	0.1407	0.0242	50.80	7.09
Test Data (Trials 5–9)	0.2482	0.0168	58.24	16.65
Training Data (Trials 1–4)	0.0589	$-1.4996\times 10^{-4}$	84.04	67.67

**Table 11:** Test Set: $L^{2}$ Position Errors (cm) for Varying Algorithm Configurations.

Config	GT Speed/GT Heading	GT Speed/Est. Heading	Est. Speed/GT Heading	Est. Speed/Est. Heading
ID	Mean (Std. Dev.)	Mean (Std. Dev.)	Mean (Std. Dev.)	Mean (Std. Dev.)
$C_{0}$	1.64 (1.13)	6.63 (6.87)	4.80 (3.82)	8.44 (7.23)
$C_{\psi }$	1.64 (1.13)	5.40 (6.37)	4.80 (3.82)	7.57 (6.88)
$C_{\psi ,z}$	1.64 (1.14)	5.40 (6.37)	4.81 (3.84)	7.57 (6.88)
$C_{\psi ,z_{ideal}}$	1.64 (1.13)	5.39 (6.35)	4.76 (3.82)	7.54 (6.87)

**Table 12:** Test Set: $L^{2}$ Position Errors (cm) for Configuration $C_{\psi ,z}$ using Est. Speed & Est. Heading.

Trial	2-min. $T_{i}$	7-min. $T_{i}$	14-min. $T_{i}$	28-min. $T_{i}$
#	Mean (Std. Dev.)	Mean (Std. Dev.)	Mean (Std. Dev.)	Mean (Std. Dev.)
Trial #5	9.23 (5.20)	10.39 (5.73)	12.83 (7.24)	14.56 (8.89)
Trial #6	9.62 (11.66)	23.36 (31.13)	40.71 (38.82)	66.68 (38.32)
Trial #7	6.10 (3.88)	10.39 (7.69)	14.90 (9.59)	16.03 (9.52)
Trial #8	6.07 (4.65)	9.39 (6.00)	8.84 (5.51)	20.31 (10.01)
Trial #9	6.73 (4.49)	11.00 (5.31)	15.70 (8.69)	37.28 (20.65)
**Average (with trial #6)**	7.55 (5.98)	12.91 (11.17)	18.60 (13.97)	30.97 (17.48)
**Average (without trial #6)**	7.03 (4.56)	10.29 (6.18)	13.07 (7.76)	22.05 (12.27)

**Table 13:** Trial #7: Algorithm runtime (s) for two-minute trajectory segments.

Process Name	Entire Trial	Avg. Time Per Segment
Feature Extraction	23.13	1.65
Madgwick Algorithm	8.79	0.63
Regression Models	0.976	0.07
Trajectory Estimation	1521.8	108.7
**Total Runtime**	1554.7 *	111.05

* Assumes the trajectory segments are optimized sequentially.

**Figure A1:**
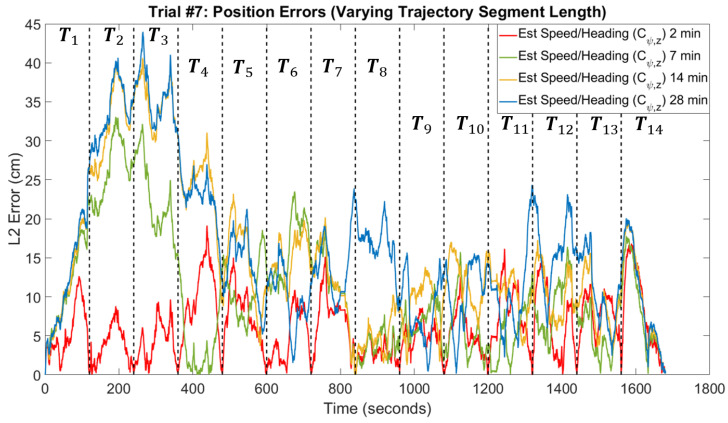
Trial #7 Position Errors (Varying Trajectory Segment Length): Distance between the ground truth and estimated position trajectories for configuration $C_{\psi ,z}$ are shown for varying trajectory segment lengths. The $T_{i}$ terms denote the ith trajectory segment for the two-minute case.

## Abbreviations

The following abbreviations are used in this manuscript:

AHRS	Attitude and Heading Reference System
GNSS	Global Navigation Satellite System
GPS	Global Positioning System
LIDAR	Light Detection and Ranging
IMU	Inertial Measurement Unit
INS	Inertial Navigation System
RADAR	Radio Detection and Ranging
SONAR	Sound Navigation and Ranging
UAV	Unmanned Aerial Vehicle
UGV	Unmanned Ground Vehicle
USAR	Urban Search and Rescue

## Appendix A. Additional Results for Trial #7

The position errors for varying trajectory segment lengths are shown for trial #7 (configuration $C_{\psi ,z}$) in Table A1 and Figure A1. Additionally, Table A2 shows the performance of the trajectory estimation algorithm in trial #7 for configuration $C_{\psi ,z}$. We see that the average across the trajectory segments is in line with the results that we see across all of the test data sets (Table 11).

**Table A1:** Trial #7: $L^{2}$ Position Errors (cm) for Configuration $C_{\psi ,z}$ using Est. Speed and Est. Heading.

Time (s)	2-min. $T_{i}$	7-min. $T_{i}$	14-min. $T_{i}$	28-min. $T_{i}$
[Start,End]	Mean (Std. Dev.)	Mean (Std. Dev.)	Mean (Std. Dev.)	Mean (Std. Dev.)
0–120	5.37 (3.33)	10.97 (5.55)	11.59 (6.44)	12.10 (6.67)
120–240	3.77 (2.33)	26.15 (3.79)	32.04 (4.61)	33.58 (3.63)
240–360	4.16 (2.48)	22.55 (4.75)	35.03 (2.79)	35.86 (3.20)
360–480	9.46 (4.76)	4.12 (4.25)	23.72 (4.73)	22.72 (3.34)
480–600	7.55 (3.50)	10.86 (3.60)	14.33 (5.19)	13.62 (4.09)
600–720	4.83 (3.21)	15.93 (4.10)	14.97 (2.68)	9.88 (3.70)
720–840	7.21 (3.43)	11.37 (4.38)	11.56 (4.73)	13.97 (3.98)
840–960	2.85 (1.26)	3.58 (1.64)	4.95 (2.39)	16.80 (2.89)
960–1080	4.87 (1.82)	6.91 (2.04)	8.91 (3.16)	8.25 (3.91)
1080–1200	5.12 (2.83)	5.61 (3.64)	12.00 (2.87)	10.46 (4.17)
1200–1320	7.42 (3.66)	4.93 (3.36)	10.32 (2.88)	10.44 (5.11)
1320–1440	6.90 (4.46)	9.02 (3.42)	10.44 (3.20)	16.19 (4.29)
1440–1560	7.64 (2.69)	5.29 (2.76)	8.89 (3.37)	10.72 (4.32)
1560–1680	8.18 (5.45)	8.17 (5.60)	9.80 (6.29)	9.79 (6.35)
**Average**	6.10 (3.23)	10.39 (3.78)	14.89 (3.95)	16.03 (4.26)

**Table A2:** Trial #7: $L^{2}$ Position Errors (cm) for Configuration $C_{\psi ,z}$—$T_{i}$’s are two minutes each.

Segment	GT Speed/GT Heading	GT Speed/Est. Heading	Est. Speed/GT Heading	Est. Speed/Est. Heading
#	Mean (Std. Dev.)	Mean (Std. Dev.)	Mean (Std. Dev.)	Mean (Std. Dev.)
1	1.29 (0.73)	1.62 (0.73)	5.37 (3.21)	5.37 (3.33)
2	2.13 (1.03)	4.01 (2.27)	3.52 (1.68)	3.77 (2.33)
3	3.19 (1.54)	3.51 (1.70)	3.49 (1.70)	4.16 (2.48)
4	1.77 (1.08)	6.59 (3.72)	3.40 (1.59)	9.46 (4.76)
5	1.10 (0.62)	5.22 (2.37)	3.34 (1.48)	7.55 (3.50)
6	1.22 (0.59)	4.71 (3.01)	1.90 (1.54)	4.83 (3.21)
7	1.42 (0.67)	3.27 (1.15)	4.48 (2.38)	7.21 (3.43)
8	1.50 (0.98)	4.02 (1.47)	2.27 (1.32)	2.85 (1.26)
9	1.31 (0.80)	4.21 (1.93)	1.67 (0.78)	4.87 (1.82)
10	1.16 (0.53)	4.55 (3.43)	2.37 (1.44)	5.12 (2.83)
11	1.36 (0.89)	5.64 (2.55)	4.75 (2.38)	7.42 (3.66)
12	1.00 (0.49)	5.48 (2.49)	4.07 (2.15)	6.90 (4.46)
13	1.92 (1.00)	7.38 (3.37)	2.66 (1.54)	7.64 (2.69)
14	1.77 (0.90)	8.32 (5.94)	1.97 (1.14)	8.18 (5.45)
**Average**	1.58 (0.85)	4.89 (2.58)	3.23 (1.74)	6.10 (3.23)

It may be noticed that the standard deviation of the position errors for trial #7 in Table 12 differs from the average standard deviations for trial #7 that are reported in Table A1. This occurs because the standard deviation in the former table is for the error over the entire trial, whereas the standard deviation in the latter table is the average of the standard deviations for each of the specified time ranges. This shows that different time ranges can have very different errors, especially as the trajectory segment length increases. More interestingly, the position errors are corrected over time using only the starting and ending ground truth states of the trajectory segments, as evidenced by the behavior of the position errors in Figure A1. An explanation for this comes from the fact that the perturbation splines are penalized when they don’t follow the speeds/headings and when the estimated end state does not match the ground truth state. As such, when the estimated position deviates from the true position—which occurs when there are erroneous speeds and/or headings—the trajectory estimation algorithm optimizes subsequent spline pieces so that future speeds and headings are followed while ensuring that the final estimated state matches the true end state. The resulting effect is that the estimated position trajectory corrects itself over time.

## References

[1] Murphy R.R., Tadokoro S., Kleiner A. (2016). Disaster robotics. Springer Handbook of Robotics.

[2] United Nations Department of Public Information 2018 Revision of World Urbanization Prospects Press Release. https://population.un.org/wup/Publications/Files/WUP2018-PressRelease.pdf.

[3] Force B. (2011). Texas Task Force 1: Urban Search and Rescue.

[4] Murphy R.R. (2004). Trial by fire [rescue robots]. IEEE Robot. Autom. Magaz..

[5] International Atomic Energy Agency (2015). The Fukushima Daiichi Accident.

[6] McKinney R., Crocco W., Stricklin K.G., Murray K.A., Blankenship S.T., Davidson R.D., Urosek J.E., Stephan C.R., Beiter D.A. (2002). Report of Investigation: Fatal Underground Coal Mine Explosions, September 23, 2001, no. 5 Mine.

[7] Lippmann M., Cohen M.D., Chen L.C. (2015). Health effects of World Trade Center (WTC) Dust: An unprecedented disaster with inadequate risk management. Crit. Rev. Toxicol..

[8] Murphy R.R. (2014). Disaster Robotics.

[9] Iida F., Ijspeert A.J. (2016). Biologically inspired robotics. Springer Handbook of Robotics.

[10] Saranli U., Buehler M., Koditschek D.E. (2001). RHex: A simple and highly mobile hexapod robot. Int. J. Robot. Res..

[11] Haldane D.W., Peterson K.C., Bermudez F.L.G., Fearing R.S. Animal-inspired design and aerodynamic stabilization of a hexapedal millirobot. Proceedings of the 2013 IEEE International Conference on Robotics and Automation.

[12] Hatazaki K., Konyo M., Isaki K., Tadokoro S., Takemura F. Active scope camera for urban search and rescue. Proceedings of the IROS 2007 IEEE/RSJ International Conference on Intelligent Robots and Systems.

[13] Glick P., Suresh S.A., Ruffatto D., Cutkosky M., Tolley M.T., Parness A. (2018). A Soft Robotic Gripper with Gecko-Inspired Adhesive. IEEE Robot. Automat. Lett..

[14] Hawkes E.W., Ulmen J., Esparza N., Cutkosky M.R. Scaling walls: Applying dry adhesives to the real world. Proceedings of the 2011 IEEE/RSJ International Conference on Intelligent Robots and Systems (IROS).

[15] Latif T. (2016). Tissue-Electrode Interface Characterization for Optimization of Biobotic Control of Roach-bots. Ph.D. Thesis.

[16] Latif T., Bozkurt A. Line following terrestrial insect biobots. Proceedings of the 2012 Annual International Conference of the IEEE Engineering in Medicine and Biology Society (EMBC).

[17] Dirafzoon A., Latif T., Gong F., Sichitiu M., Bozkurt A., Lobaton E. Biobotic motion and behavior analysis in response to directional neurostimulation. Proceedings of the 2017 IEEE International Conference on Acoustics, Speech and Signal Processing (ICASSP).

[18] van Casteren A., Codd J.R. (2010). Foot morphology and substrate adhesion in the Madagascan hissing cockroach, Gromphadorhina portentosa. J. Insect Sci..

[19] Jayaram K., Full R.J. (2016). Cockroaches traverse crevices, crawl rapidly in confined spaces, and inspire a soft, legged robot. Proc. Natl. Acad. Sci. USA.

[20] Bozkurt A., Lobaton E., Sichitiu M. (2016). A biobotic distributed sensor network for under-rubble search and rescue. Computer.

[21] Latif T., Bozkurt A. (2017). Roach Biobots: Toward Reliability and Optimization of Control. IEEE Pulse.

[22] Dirafzoon A., Bozkurt A., Lobaton E. (2017). A framework for mapping with biobotic insect networks: From local to global maps. Robot. Autonom. Syst..

[23] Groves P.D. (2013). Principles of GNSS, Inertial, and Multisensor Integrated Navigation Systems.

[24] Scaramuzza D., Fraundorfer F. (2011). Visual odometry [tutorial]. IEEE Robot. Autom. Mag..

[25] Bloesch M., Omari S., Hutter M., Siegwart R. Robust visual inertial odometry using a direct EKF-based approach. Proceedings of the 2015 IEEE/RSJ International Conference on Intelligent Robots And Systems (IROS).

[26] Li M., Mourikis A.I. (2013). High-precision, consistent EKF-based visual-inertial odometry. Int. J. Robot. Res..

[27] Leutenegger S., Lynen S., Bosse M., Siegwart R., Furgale P. (2015). Keyframe-based visual–inertial odometry using nonlinear optimization. Int. J. Robot. Res..

[28] Wooden D., Malchano M., Blankespoor K., Howardy A., Rizzi A.A., Raibert M. Autonomous navigation for BigDog. Proceedings of the 2010 IEEE International Conference on Robotics and Automation.

[29] Kuutti S., Fallah S., Katsaros K., Dianati M., Mccullough F., Mouzakitis A. (2018). A survey of the state-of-the-art localization techniques and their potentials for autonomous vehicle applications. IEEE Int. Things J..

[30] Yurtsever E., Lambert J., Carballo A., Takeda K. (2019). A survey of autonomous driving: Common practices and emerging technologies. arXiv.

[31] Adams M., Adams M.D., Jose E. (2012). Robotic Navigation and Mapping With Radar.

[32] Cornick M., Koechling J., Stanley B., Zhang B. (2016). Localizing ground penetrating radar: A step toward robust autonomous ground vehicle localization. J. Field Robot..

[33] Paull L., Saeedi S., Seto M., Li H. (2013). AUV navigation and localization: A review. IEEE J. Ocean. Eng..

[34] Panish R., Taylor M. (2011). Achieving high navigation accuracy using inertial navigation systems in autonomous underwater vehicles. OCEANS 2011 IEEE-Spain.

[35] Harle R. (2013). A survey of indoor inertial positioning systems for pedestrians. IEEE Commun. Surv. Tutor..

[36] Skog I., Handel P., Nilsson J.O., Rantakokko J. (2010). Zero-velocity detection—An algorithm evaluation. IEEE Trans. Biomed. Eng..

[37] Wahlström J., Skog I., Gustafsson F., Markham A., Trigoni N. (2019). Zero-velocity detection—A Bayesian approach to adaptive thresholding. IEEE Sens. Lett..

[38] Cortés S., Solin A., Kannala J. Deep learning based speed estimation for constraining strapdown inertial navigation on smartphones. Proceedings of the 2018 IEEE 28th International Workshop on Machine Learning for Signal Processing (MLSP).

[39] Wagstaff B., Kelly J. LSTM-based zero-velocity detection for robust inertial navigation. Proceedings of the 2018 International Conference on Indoor Positioning and Indoor Navigation (IPIN).

[40] Kone Y., Zhu N., Renaudin V., Ortiz M. (2020). Machine Learning-Based Zero-Velocity Detection for Inertial Pedestrian Navigation. IEEE Sens. J..

[41] Shu Y., Shin K.G., He T., Chen J. Last-mile navigation using smartphones. Proceedings of the 21st Annual International Conference on Mobile Computing And Networking.

[42] Hannink J., Kautz T., Pasluosta C.F., Barth J., Schülein S., Gaßmann K.G., Klucken J., Eskofier B.M. (2017). Mobile stride length estimation with deep convolutional neural networks. IEEE J. Biomed. Health Inform..

[43] Schmidt G.T. (2015). Navigation sensors and systems in GNSS degraded and denied environments. Chin. J. Aeron..

[44] El-Sheimy N., Chiang K.W., Noureldin A. (2006). The utilization of artificial neural networks for multisensor system integration in navigation and positioning instruments. IEEE Trans. Instrum. Meas..

[45] Semeniuk L., Noureldin A. (2006). Bridging GPS outages using neural network estimates of INS position and velocity errors. Meas. Sci. Technol..

[46] Adusumilli S., Bhatt D., Wang H., Bhattacharya P., Devabhaktuni V. (2013). A low-cost INS/GPS integration methodology based on random forest regression. Expert Syst. Appl..

[47] Adusumilli S., Bhatt D., Wang H., Devabhaktuni V., Bhattacharya P. (2015). A novel hybrid approach utilizing principal component regression and random forest regression to bridge the period of GPS outages. Neurocomputing.

[48] Jolliffe I.T. (1982). A note on the use of principal components in regression. J. R. Stat. Soc. Ser. C Appl. Stat..

[49] Zhang Y. (2019). A Fusion Methodology to Bridge GPS Outages for INS/GPS Integrated Navigation System. IEEE Access.

[50] Esfahani M.A., Wang H., Wu K., Yuan S. (2019). AbolDeepIO: A novel deep inertial odometry network for autonomous vehicles. IEEE Trans. Intell. Transp. Syst..

[51] Chen C., Lu X., Markham A., Trigoni N. Ionet: Learning to cure the curse of drift in inertial odometry. Proceedings of the Thirty-Second AAAI Conference on Artificial Intelligence.

[52] Brossard M., Barrau A., Bonnabel S. (2019). RINS-W: Robust inertial navigation system on wheels. arXiv.

[53] Brossard M., Barrau A., Bonnabel S. (2019). AI-IMU dead-reckoning. arXiv.

[54] Silva do Monte Lima J.P., Uchiyama H., Taniguchi R.i. (2019). End-to-End Learning Framework for IMU-Based 6-DOF Odometry. Sensors.

[55] Zhang H., Li T., Yin L., Liu D., Zhou Y., Zhang J., Pan F. (2019). A Novel KGP Algorithm for Improving INS/GPS Integrated Navigation Positioning Accuracy. Sensors.

[56] Li J., Song N., Yang G., Li M., Cai Q. (2017). Improving positioning accuracy of vehicular navigation system during GPS outages utilizing ensemble learning algorithm. Inform. Fus..

[57] Yan H., Herath S., Furukawa Y. (2019). RoNIN: Robust Neural Inertial Navigation in the Wild: Benchmark, Evaluations, and New Methods. arXiv.

[58] Liu W., Caruso D., Ilg E., Dong J., Mourikis A., Daniilidis K., Kumar V., Engel J., Valada A., Asfour T. (2020). TLIO: Tight Learned Inertial Odometry. IEEE Robot. Autom. Lett..

[59] Groves P.D. (2013). The PNT boom: Future trends in integrated navigation. Inside GNSs.

[60] Groves P.D., Wang L., Walter D., Martin H., Voutsis K., Jiang Z. The four key challenges of advanced multisensor navigation and positioning. Proceedings of the 2014 IEEE/ION, Position, Location and Navigation Symposium-PLANS 2014.

[61] Cole J., Mohammadzadeh F., Bollinger C., Latif T., Bozkurt A., Lobaton E. (2017). A study on motion mode identification for cyborg roaches. Proceedings of the 2017 IEEE International Conference on Acoustics, Speech and Signal Processing (ICASSP).

[62] Madgwick S.O., Harrison A.J., Vaidyanathan R. Estimation of IMU and MARG orientation using a gradient descent algorithm. Proceedings of the 2011 IEEE International Conference on Rehabilitation Robotics.

[63] Kirk D.E. (2004). Optimal Control Theory: An Introduction.

[64] Kincaid D., Kincaid D.R., Cheney E.W. (2009). Numerical Analysis: Mathematics of Scientific Computing.

[65] Dubins L.E. (1957). On curves of minimal length with a constraint on average curvature, and with prescribed initial and terminal positions and tangents. Amer. J. Math..

[66] Reeds J., Shepp L. (1990). Optimal paths for a car that goes both forwards and backwards. Pac. J. Math..

[67] MATLAB (2018). Statistics and Machine Learning Toolbox Version 11.3: MATLAB Release 2018a.

[68] Kuipers J.B. (1999). Quaternions and Rotation Sequences.

[69] Cole J., Agcayazi T., Latif T., Bozkurt A., Lobaton E. Speed estimation based on gait analysis for biobotic agents. Proceedings of the 2017 IEEE SENSORS.

[70] Breiman L. (2001). Random forests. Mach. Learn..

[71] Loh W.Y. (2011). Classification and regression trees. Wiley Int. Revi. Data Min. Knowl. Discov..

[72] Loh W.Y. (2014). Fifty years of classification and regression trees. Int. Stat. Rev..

[73] Breiman L., Friedman J., Stone C.J., Olshen R.A. (1984). Classification and rEgression Trees.

[74] Zhang C., Ma Y. (2012). Ensemble Machine Learning: Methods and Applications.

[75] Hastie T., Tibshirani R., Friedman J. (2009). The eLements of Statistical Learning: Data Mining, Inference, and Prediction.

[76] MATLAB (2018). Optimization Toolbox Version 8.1: MATLAB Release 2018a.

[77] Nocedal J., Wright S. (2006). Numerical Optimization.

[78] Xiong H., Agcayazi T., Latif T., Bozkurt A., Sichitiu M.L. Towards acoustic localization for biobotic sensor networks. Proceedings of the 2017 IEEE SENSORS.

[79] Canny J. (1986). A computational approach to edge detection. IEEE Trans. Patt. Anal. Mach. Intell..

